# How Memory Switches Brain Responses of Patients with Post-traumatic Stress Disorder

**DOI:** 10.1093/texcom/tgab021

**Published:** 2021-03-20

**Authors:** Jun Inoue, Kayako Matsuo, Toshiki Iwabuchi, Yasuo Takehara, Hidenori Yamasue

**Affiliations:** 1 Department of Child and Adolescent Psychiatry, Hamamatsu University School of Medicine, Hamamatsu City, Shizuoka 431-3192, Japan; 2 Center for Preventive Medicine in Mental Health, Department of Psychiatry, Hamamatsu University School of Medicine, Hamamatsu City, Shizuoka 431-3192, Japan; 3 Research Center for Child Mental Development, Hamamatsu University School of Medicine, Hamamatsu City, Shizuoka 431-3192, Japan; 4 United Graduate School of Child Development, Hamamatsu University School of Medicine, Hamamatsu city, Shizuoka 431-3192, Japan; 5 Department of Radiology, Hamamatsu University Hospital, Hamamatsu city, Shizuoka, Japan 431-3192; 6 Department of Psychiatry, Hamamatsu University School of Medicine, Hamamatsu city, Shizuoka 431-3192, Japan; 7 Center for Research Collaboration and Support, Dokkyo Medical University School of Medicine, Mibu-machi, Shimotsuga-gun, Tochigi 321-0293, Japan; 8 Department of Fundamental Development for Advanced Low Invasive Diagnostic Imaging, Graduate School of Medicine, Nagoya University, Nagoya City, Aichi 464-8601, Japan

**Keywords:** eye movement desensitization and reprocessing (EMDR), functional magnetic resonance imaging (fMRI), hippocampus, post-traumatic stress disorder (PTSD), script-driven imagery task

## Abstract

To characterize the brain responses to traumatic memories in post-traumatic stress disorder (PTSD), we conducted task-employed functional magnetic resonance imaging and, in the process, devised a simple but innovative approach—correlation computation between task conditions. A script-driven imagery task was used to compare the responses with a script of the patients’ own traumatic memories and with that of tooth brushing as a daily activity and to evaluate how eye movement desensitization and reprocessing (EMDR), an established therapy for PTSD, resolved the alterations in patients. Nine patients with PTSD (seven females, aged 27–50 years) and nine age- and gender-matched healthy controls participated in this study. Six patients underwent the second scan under the same paradigm after EMDR. We discovered intense negative correlations between daily and traumatic memory conditions in broad areas, including the hippocampus; patients who had an intense suppression of activation during daily recognition showed an intense activation while remembering a traumatic memory, whereas patients who had a hyperarousal in daily recognition showed an intense suppression while remembering a traumatic memory as a form of “shut-down.” Moreover, the magnitude of the discrepancy was reduced in patients who remitted after EMDR, which might predict an improved prognosis of PTSD.

## Introduction

Post-traumatic stress disorder (PTSD) occurs after exposure to an extraordinarily dreadful experience and is characterized by symptoms of hyperarousal, avoidance of situations similar to the original experience, and memory intrusions into daily life (American Psychiatric Association 2013). Accumulating data indicate that PTSD alters patients’ brains (Yamasue et al. 2003; Kasai et al. 2008; Akiki et al. 2017; Malejko et al. 2017; Fenster et al. 2018; Mary et al. 2020). However, the results have not been consistent among studies (Shin et al. 2006; Akiki et al. 2017; Malejko et al. 2017). Moreover, PTSD also manifests a considerable heterogeneity in treatment responses (Bradley et al. 2005; Schottenbauer et al. 2008; Murphy and Smith 2018), which might partly reflect the heterogeneity in brain responses. If it were the case, we may have been able to find a brain activity marker for good treatment responses (Dickie et al. 2011; Malejko et al. 2017).

**Figure 1:**
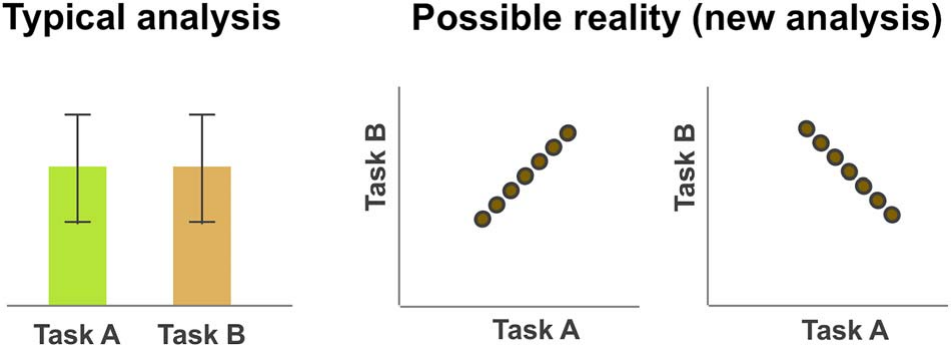
Illustration of the analysis for task-employed fMRI. Most fMRI studies typically compare the signal estimate magnitudes between employed tasks in a voxel-based manner. The left panel shows an example where there would appear to be no significant differences between tasks A and B; both tasks induced similar intensities of activation at that brain area on average. However, the data of the two tasks may have a hidden relationship with a positive or negative correlation as shown in the right panel; individuals (indicated as circles in the right panel) with more intense activation during task A could also have more intense activation during task B than other individuals (i.e., a positive correlation), or conversely, less intense activation during the task B (i.e., a negative correlation). Such correlations cannot be detected by using the typical analysis methods, as in the left panel.

Therefore, using functional magnetic resonance imaging (fMRI), we aimed to find an association between brain activation and PTSD symptoms with a potential prognostic value. The considerable heterogeneity mentioned above challenged us to reconsider the existing analysis frameworks instead of continuing with the conventional methodology. In the process of brain activity observation, in which we incidentally found both positive and negative correlations with certain psychological subscale scores, we developed a simple but innovative method of computing brain activity correlation estimates between task conditions. To our knowledge, this is the first application of correlation analysis to simply and directly examine the associations between conditions arranged in task-employed fMRI, which is noteworthy because we typically compare the magnitudes of activity estimates but overlook the possibility of such correlations between tasks (Fig. 1). Moreover, PTSD would be the best applicable disorder for this new method because patients with PTSD often show two alternating extremes, including hyper- and hypoarousals (Ogden and Minton 2000; Corrigan et al. 2011). These alterations may be due to alternating brain conditions that could be reflected in a negative correlation between activity estimates. By conducting a second scan using the same task paradigm, we also investigated changes after treatment with eye movement desensitization and reprocessing (EMDR) (Shapiro 2014). EMDR has been recognized as one of the most effective PTSD treatments (van der Kolk et al. 2007; Lee and Cuijpers 2013; van den Berg et al. 2015).

For the task paradigm, we applied a previously established script-driven imagery task (Rauch et al. 1996; Lanius et al. 2001, 2002, 2003, 2005; Supplementary Table S1). Participants listened to a narration and were subsequently asked to recall it. In one condition, the narration spoke about a harmless daily common activity, whereas in the other, it dealt with the patient’s own traumatic episode. The two conditions allowed us to deliberately compare two types of brain responses in patients: response to a harmless daily event and to a harmful traumatic memory. These two response types would involuntarily switch from one to the other. Using this paradigm, we aimed to characterize the brain activity patterns behind these two response types that might reflect the prognostic features in PTSD.

## Materials and Methods

### Participants

**Figure 2:**
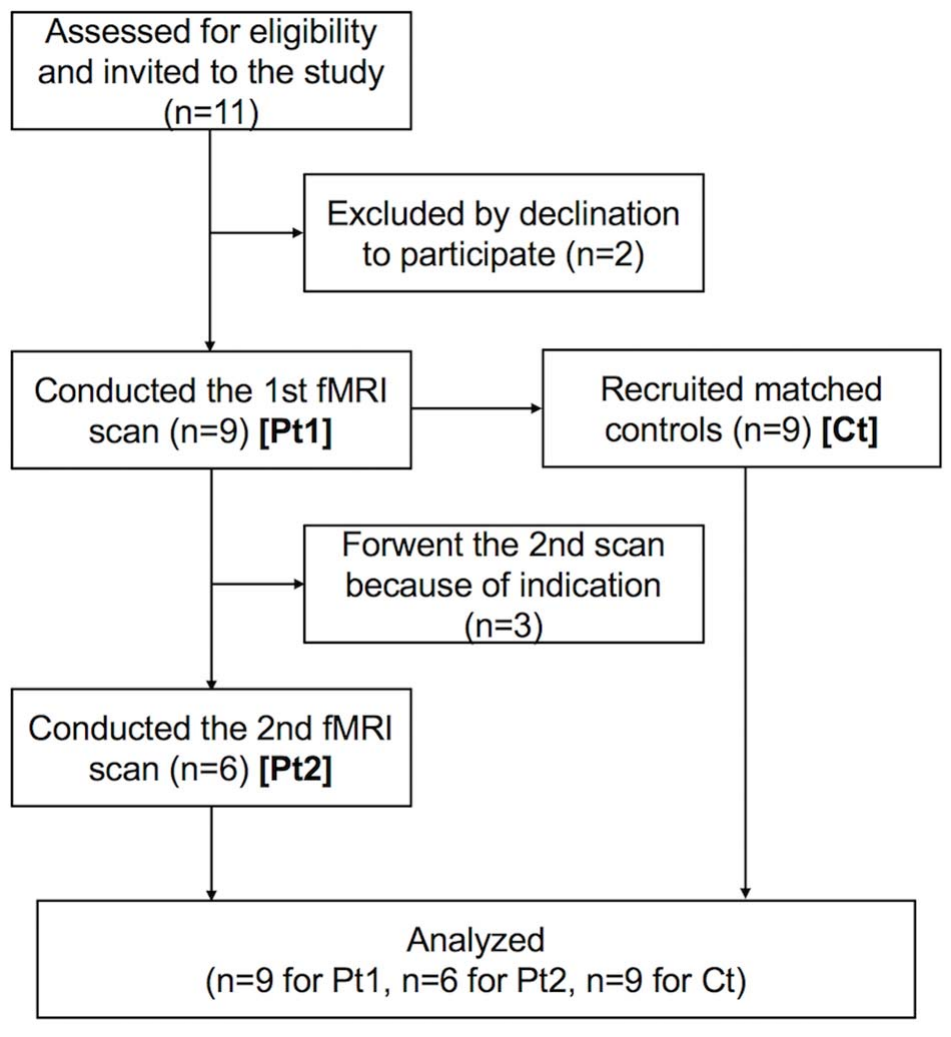
Diagram of the research progress. This diagram was prepared by referring to the study by Moher et al. (2010). More detailed information is provided in the Supplementary Material (p. S3).

Nine patients with PTSD and age- and gender-matched normal controls participated in this study (Fig. 2; Table 1). All participants provided written informed consent, and the protocol adhered to the Declaration of Helsinki and was approved by the institutional review board of Hamamatsu University School of Medicine. All patients were inpatients or outpatients of Hamamatsu University Hospital, Hamamatsu, Japan, who met the criteria of the Diagnostic and Statistical Manual of Mental Disorders, Fifth Edition (DSM-5) for PTSD. Table 1 summarizes the participant characteristics (see Supplementary Tables S2–S4 for more details). Six out of the nine patients attended the second scan after EMDR. For the remaining three patients (patient identification numbers [IDs] 7–9), we forwent beginning the standard EMDR protocol during the study period based on the guidelines for severe PTSD (International Society for the Study of Trauma and Dissociation 2011) (these patients were labeled as “severe”). Patients with IDs 4–6, whose final scores of Subjective Units of Disturbance (SUD) Scale (see EMDR procedure) did not decline to 0 or 1, discontinued the treatment because of financial reasons and/or physical move (“discontinued”). By contrast, patients with IDs 1–3 recovered well, lowering their SUD to 0 or 1 (“remitted”). No drug washout occurred before the scanning. Controls were recruited from the medical staff in our institute or from other clinics nearby. We named the data from the patients’ first scan “Pt1,” the data from the second “Pt2,” and from the controls “Ct.”

**Table 1:** Overview of participant characteristics

Table 1-1. Demographic data									
	Gender	Age (years)	Handedness	Education (years)	SUD 1	Times EMDR	Interval (days)	SUD 2	
Patients	F: 7, M: 2	32.8 (7.0)	69.8 (64.0)	13.9 (2.3)	7.9 (2.1)	8.7 (2.9)	339.3 (111.4)	2.8 (2.8)	
Controls	F: 7, M: 2	31.4 (7.4)	100 (0)	18.3 (0.9)	—	—	—	—	
Table 1-2. Diagnostic indices									
	CAPS				IES-R				DES-II
	Ree	Avo and Num	Hyp	Total	Int	Avo	Hyp	Total	
Pt1	24.4 (7.9)	33.4 (10.3)	22.9 (8.1)	80.8 (20.1)	22.8 (6.0)	16.7 (5.5)	16.7 (4.9)	56.1 (13.5)	950.0 (575.6)
Pt2	9.2 (5.1)	21.3 (15.4)	16.7 (8.3)	47.2 (25.9)	9.8 (5.3)	15.0 (8.0)	11.0 (5.4)	35.8 (15.9)	533.3 (437.6)
Table 1-3. RSDI scores									
ID	Ree	Avo	Dis	Total					
Pt1	16.4 (8.1)	8.6 (5.9)	5.9 (6.3)	30.9 (9.9)					
Pt2	11.3 (5.9)	4.2 (5.5)	2.2 (2.7)	17.7 (12.3)					
Ct	5.4 (4.7)	1.2 (2.0)	0.8 (1.7)	7.4 (7.7)					

Note: Frequency counts are shown for gender, whereas means (SDs) are shown for all other items. Age, age at the time of the first scan; handedness, Edinburgh Handedness Inventory score (Oldfield 1971); SUD 1, SUD score at the time of the first scan; Times EMDR, session number for EMDR (combined) in phases 4–7 conducted between the first and the second scans; Interval, number of days between the first and second scans; SUD 2, SUD score at the time of the second scan; F, female; M, male; Pt1, patients at the first scan; Pt2, patients at the second scan; Ct, controls; Ree, reexperiencing; Avo, avoidance; Num, numbing; Hyp, hyperarousal; Int, intrusions; Dis, dissociation. Additional information can be found in Supplementary Tables S2–S4.

### Image Data Acquisition

Image data were acquired using a 3-Tesla MRI (Discovery MR750 3.0T; General Electric Healthcare) with a 32-channel phased array head coil. Functional images were acquired by a pulse sequence to detect the blood oxygenation level–dependent (BOLD) T2* signals using the following parameters: time repetition (TR), 2000 ms; time echo (TE), 22 ms; flip angle, 90°; field of view (FOV), 192 mm; matrix, 64 × 64; number of slices, 45 axial-oblique slices covering the whole brain; slice thickness, 3 mm without interslice space; slice acquisition order, interleaved; and volumes, 270 after 4 volumes of additional dummy data acquisition. T1-weighted images were also acquired as anatomical references by a sequence of T1 (3D time-of-flight fast spoiled gradient echo brain volume imaging; 3DFSPGR BRAVO) using the following parameters: TR, 8.2 ms; TE, 3.2 ms; flip angle, 12°; inversion time, 450 ms; FOV, 256 mm; and matrix, 256 × 256. The images were reconstructed into 170 slices with a slice thickness of 1 mm.

### fMRI Paradigm

We followed a previously developed paradigm of a script-driven imagery task (Rauch et al. 1996; Lanius et al. 2002, 2005) with minor modifications. Two task runs, Tooth task and Trauma task, in this order, were conducted. A resting-state run preceded the two task runs (not reported here). Both tasks had the same block-design structure, including four kinds of blocks in the following order: fixation, narration, remembering, and breathing (Supplementary Fig. S1). A block set was repeated three times (total 9 min per run). The fixation block displayed a plus mark in the middle of the monitor for 1 min. Participants watched the fixation while thinking of nothing in particular. The narration block presented a description of an event both orally and visually in text form for 30 s. For the stimuli, a read-aloud of the text displayed in the monitor was prepared in advance (read by J.I.). In the Tooth task, the description was of an episode of tooth brushing as a daily activity. In the Trauma task, the description was of the patients’ own traumatic experience. Before the scanner session, during clinical interviews, the patients and the author (J.I.) jointly selected an episode from each individual’s traumatic events for use in fMRI. Since every run consisted of three block repetitions, the text was repeated three times in a run. We matched the average number of mora (i.e., the phonological unit in Japanese) in the Trauma narrations across patients (mean, 213.8; standard deviation [SD], 8.5; range, 199–224) to the number of mora in the Tooth narration (215) to make the conditions equivalent. Their matched control viewed and heard an identical description to that viewed and heard by his or her counterpart. The next paradigm block, the remembering block, presented an instruction on the monitor to continue remembering the episode for another 30-s period. Finally, the breathing block asked participants to breathe deeply (literally, “breathe slowly” in Japanese) for 1 min. Oral instructions were provided at the beginning of the breathing block, while the text (the same content as that delivered orally) remained on the monitor throughout the block. The same paradigm from the first scan was repeated in the post-treatment scan. Please refer to the Supplementary Material for more information regarding the task paradigm.

### Procedure

fMRI stimuli were displayed using E-Prime2 software (Psychology Software Tools, Inc.) through MRI compatible equipment (VisuaStim Digital, Resonance Technology, Inc.). Participants’ vision was normal or was corrected to normal by lenses attached to goggles through which the stimuli were viewed. They wore headphones to receive the auditory stimulation. Patients received a brief explanation of the paradigm at the time of providing informed consent in addition to a full explanation before the scan. All participants also practiced once before entering the scanner suite.

Heart rate and breathing rate were monitored during functional imaging using the MRI equipment of a fiber-optic device placed over the index finger for heart rates and using a pneumatic belt around the abdomen for breathing rates. See Supplementary Material for an analysis of these physiological data (Supplementary Table S5).

### Psychological Assessments

All participants rated their emotional responses during the Trauma task immediately after the scanner session using a Japanese translation of the Responses to Script-Driven Imagery (RSDI) Scale (Hopper, Frewen, Sack, et al. 2007; Hopper, Frewen, van der Kolk, et al. 2007). RSDI is a self-scoring assessment to evaluate the aspects of reexperiencing, avoidance, and dissociation during a script-driven imagery task. Additionally, all patients underwent comprehensive psychological assessments, including Japanese versions of the Clinician-Administered PTSD Scale (CAPS) (Blake et al. 1995; Asukai et al. 2003), the Impact of Event Scale-Revised (IES-R-J) (Asukai et al. 2002; Weiss 2004), and the Dissociative Experience Scale-II (DES-II) (Carlson and Putnam 1993; Tanabe 1994). Patients underwent these assessments again by the time of the second scan. See Supplementary Table S6 for the improvements in the scores after EMDR.

### EMDR Procedure

EMDR treatments were administered according to a standard protocol (Shapiro 1995, 2001). Patients talked about what they experienced after pursuing the therapist’s finger going right and left while focusing on the traumatic memory. The protocol involved time-to-time assessments of the subjective intensity of disturbance ranging from 0 to 10 (i.e., SUD; Supplementary Table S2). A more detailed description of the EMDR procedure is found in the Supplementary Material.

### Image Analysis

MR images were analyzed using the statistical parametric mapping (SPM) software SPM12 (Wellcome Trust Centre for Neuroimaging, University College London) and MATLAB (The MathWorks, Inc.). Functional images were first corrected for slice timing, spatially realigned to the first volume, coregistered to the skull-stripped T1-weighted image, spatially normalized using the T1-weighted image, and smoothed (full width at half maximum of 6 mm isotropic). Condition-specific contrasts were individually estimated with a design matrix, including the four block conditions of both Tooth and Trauma task runs. Individual realignment parameters (six dimensions) were also entered. We computed the following contrast estimates for each run: narration versus fixation (Nar) and remembering versus fixation (Rem). The breathing blocks were disregarded during contrast estimation to avoid the influence of head motions during agitated breathing. As a result, we obtained the following four types of individual contrast estimates: Tooth Nar, Tooth Rem, Trauma Nar, and Trauma Rem. Tooth Nar represented recognition of a daily activity; Tooth Rem, activity remembrance; Trauma Nar, recognition of a traumatic event of the patient; and Trauma Rem, event remembrance.

We first used conventional random-effects group statistics to estimate the group average effects using the individual contrast estimates. We adopted a repeated-measures analysis of variance (ANOVA) with three within-factors: group (Pt1 and Ct), task (Tooth and Trauma), and condition (Nar and Rem). We had to exclude Pt2 because of the lack of data from “severe” patients and the small sample size (Friston et al. 1999; Carter et al. 2008). Maps were generated at multiple thresholds (*P* < 0.001, *P* < 0.005, and *P* < 0.05; uncorrected for multiple comparisons) to observe the overall picture.

### New Analysis Method: Correlation Computation

Originally, we next aimed to examine the relationships of the psychological assessment scores with neural activities; using the results of a regression analysis with the assessment scores, we performed a series of region-of-interest (ROI) analyses (details in Supplementary Material). During these ROI analyses, we discovered that one of the patients’ subscale scores had a positive correlation with the average contrast estimate (ACE) in an ROI of a task condition but a negative correlation with that of another condition. This implied that the brain activities in the two conditions were negatively correlated with each other. To investigate further, we then computed the correlation coefficients among the ACEs themselves (instead of between ACEs and the assessment scores above) for all contrast pairs from Tooth Nar, Tooth Rem, Trauma Nar, and Trauma Rem. Heatmaps were made to visualize the correlation matrices.

As we observed interesting positive and negative correlation patterns in these ROI-based correlation matrices, we then computed voxel-by-voxel correlation coefficients between each contrast pair to make a correlation map of the brain. For this voxel-based correlation map, we applied the Spearman’s rank correlation method to minimize the effect of outliers. More specifically, we took contrast estimate values at the same brain coordinates from a pair, computed the Spearman’s rank correlation coefficient, and used the coefficient as the voxel value at those coordinates. We finally obtained six volumes in total for each participant group.

As we found intense and widespread negative correlations in the correlation maps of Pt1 between Tooth Nar and Trauma Rem as well as between Tooth Rem and Trauma Rem, we further examined these particular pairs. We specifically focused on the ROIs at the hippocampus (HP) as well as the primary auditory cortex (A1) because of their intense negative correlations between conditions (see Results). We individually extracted the contrast estimate values to average within the sphere (20-mm diameter around the center coordinates) for Tooth Nar, Tooth Rem, and Trauma Rem. We computed absolute difference in the estimates between Tooth Nar and Trauma Rem as well as between Tooth Rem and Trauma Rem as the parameter of the magnitude of discrepancy between the conditions; we then compared them using one-way ANOVA or *t*-test among subject groups as well as among the symptom-based classification of remitted, discontinued, and severe.

## Results

### Standard SPM Analysis

Whole-brain ANOVA by SPM demonstrated increased activity in regions belonging to the default mode network (DMN), including the medial prefrontal cortex (mPFC) and the lateral parietal cortex in Pt1 relative to Ct, specifically during the Trauma task (Supplementary Fig. S2; Supplementary Table S7). By contrast, compared with Ct, Pt1 exhibited a decreased activity in the primary visual cortex (V1), specifically during the Tooth task (Supplementary Fig. S2).

### Correlation Analyses

**Figure 3:**
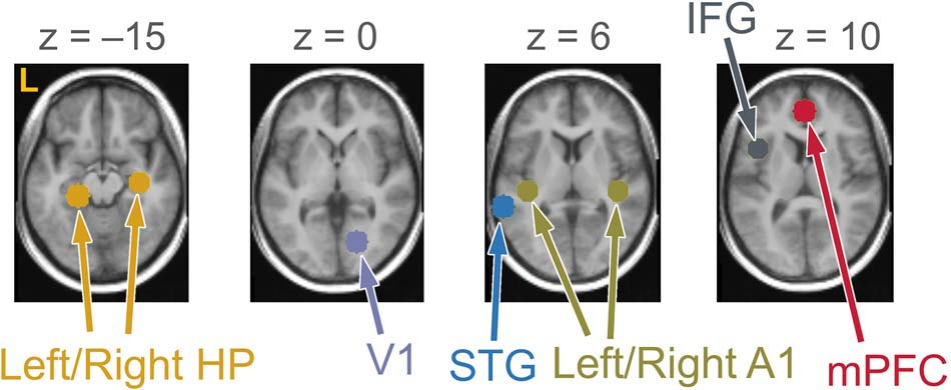
Locations of ROIs superimposed onto axial sections of average T1-weighted images. The left side of the maps represents the left side of the brain (L). STG, superior temporal gyrus; IFG, inferior frontal gyrus; *z*, *z*-coordinate. Center coordinates: left HP [−22, −30, −14]; right HP [34, −18, −16]; mPFC [0, 46, 10]; left A1 [−40, −26, 4]; right A1 [42, −26, 8]; V1 [18, −72, 0]; IFG [−42, 12, 12]; STG [−62, −40, 6].

**Figure 4:**
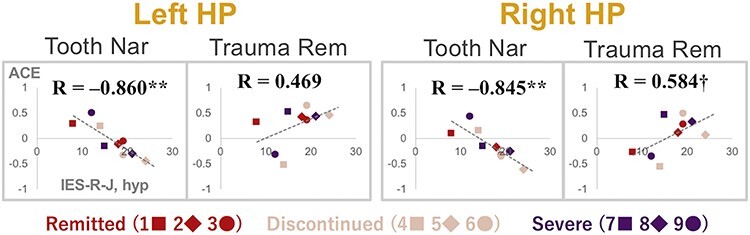
Scatter diagrams showing the relationships between ACE and IES-R-J hyperarousal subscale (hyp) score at left and right HP ROIs. The horizontal axis shows the IES-R-J hyp score, whereas the vertical axis shows the ACE at the left or right HP for Tooth Nar or Trauma Rem. Plots represent patients at the first scan (Pt1) as indicated in the bottom part; numbers 1–9 correspond to IDs in Supplementary Table S2. Equations indicate Pearson’s product–moment correlation coefficients between the subscale score and ACE. **, *P* < 0.01; †, *P* < 0.10.

The analysis with eight ROIs (Fig. 3; details in Supplementary Table S8; *T* ≥ 3.55 for peak coordinates; ANOVA results in Supplementary Table S9) revealed that the ACEs in ROIs were generally positively correlated with assessment scores; that is, activities escalated according to the increase in assessment scores (Supplementary Figs S3 and S4). However, we discovered that some ROIs had both negative and positive correlations with a subscale score; specifically, the left and right HPs showed a negative correlation with the hyperarousal subscale of IES-R-J in Pt1 during Tooth Nar (Pearson’s product–moment correlation coefficient [*R*] = −0.860 and − 0.845 for left and right HPs, respectively; *P* < 0.01 for both; *n* = 9), while there was a positive correlation with the same subscale during Trauma Rem (*R* = 0.469 and 0.584, *P* > 0.10 and *P* < 0.10, respectively; *n* = 9) (Fig. 4).

**Figure 5:**
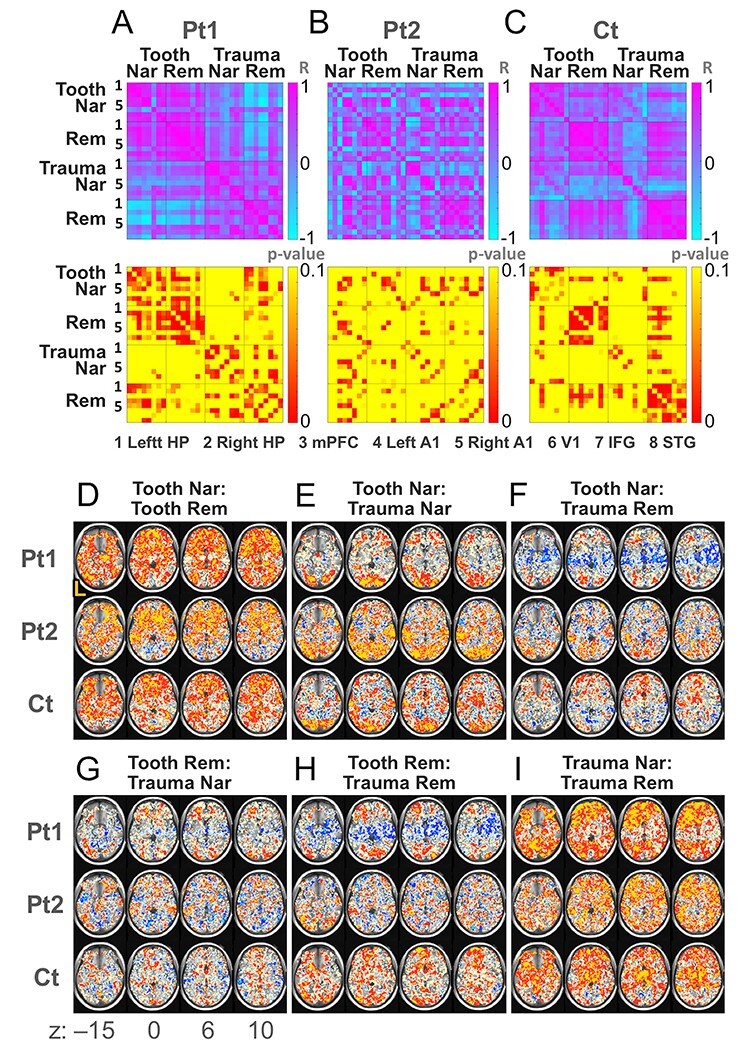
Correlations among contrast estimates. (*A*) Correlation matrices of ROI-based correlation coefficients between the ACEs (upper panel) and the corresponding *P* values (lower panel) for Pt1, (*B*) Pt2, and (*C*) Ct. Left-hand numbers (only 1 and 5 are visible) correspond to 1) ROIs of the left hippocampus (HP), 2) right HP, 3) mPFC, 4) left A1, 5) right A1, 6) V1, 7) left IFG, and 8) left STG. Numbers in the right side of matrices indicate Pearson’s product–moment correlation coefficients (*R*) for upper matrices and the *P* values for lower matrices. (*D*) Correlation maps of voxel-based correlation coefficients between Tooth Nar and Tooth Rem, (*E*) Tooth Nar and Trauma Nar, (*F*) Tooth Nar and Trauma Rem, (*G*) Tooth Rem and Trauma Nar, (*H*) Tooth Rem and Trauma Rem, and (*I*) Trauma Nar and Trauma Rem. The left side of the maps represents the left side of the brain (L). Axial sections at *z*-coordinates of −15, 0, 6, and 10 in the Montreal Neurological Institute (MNI) spacee. Mustard color indicates voxels whose Spearman’s rank correlation coefficients are ≥0.8; red, ≥0.5; cream, ≥0.2; cyan, ≤− 0.8; blue, ≤− 0.5; and ice blue, ≤− 0.2.

We also found that correlations of the ACEs with each other demonstrated specific patterns of subject groups (Fig. 5*A*–*C*). Pt1 generally showed positive correlations within the same task (i.e., Tooth or Trauma), but increased negative correlations between tasks (Fig. 5*A*). Contrastingly, Pt2 showed a mixture of positive and negative correlations regardless of task differences (Fig. 5*B*). Moreover, Ct showed positive correlations within the Tooth Rem as well as within the Trauma Rem (Fig. 5*C*). See Supplementary Material for an examination using Jennrich’s test (Jennrich 1970) and ANOVA for the objective validation of this observation (Supplementary Fig. S5; Supplementary Table S10).

### Correlation Map and Magnitude of Discrepancy

**Figure 6:**
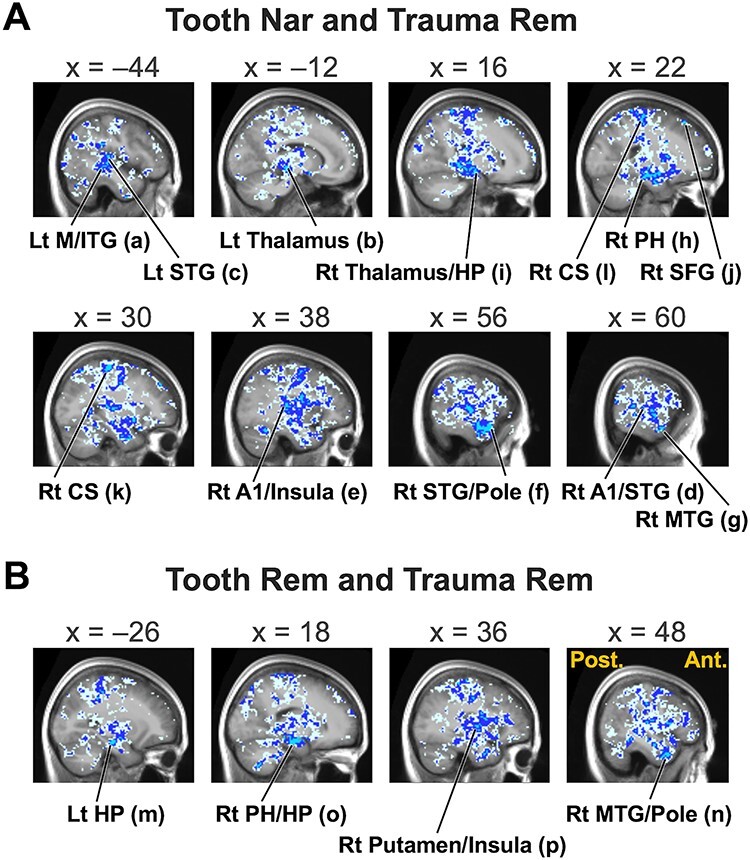
Sagittal sections showing top negative correlations between Tooth Nar and Trauma Rem (*A*) as well as between Tooth Rem and Trauma Rem (*B*) in Pt1. *x*-coordinates correspond to the MNI space. Post., posterior; Ant., anterior; Lt, left; Rt, right. Labels (a)–(p) correspond to those in Table 2. Cyan color indicates voxels whose Spearman’s rank correlation coefficients are ≤− 0.8; blue, ≤− 0.5; and ice blue, ≤− 0.2.

The voxel-wise correlation maps showed intense negative correlations between Tooth Nar/Rem and Trauma Rem, specifically in the HP, the parahippocampal gyrus (PH), the amygdala, the insula, and other temporal structures in Pt1 (Fig. 5*F*,*H*; Table 2; Fig. 6). By contrast, positive correlations prevailed in Ct (Fig. 5*H*), except for some regions, including the V1 (Fig. 5*F*). Positive correlations also prevailed between Tooth Nar and Tooth Rem, as well as between Trauma Nar and Trauma Rem, for all participant groups (Fig. 5*D*,*I*). Among the eight ROIs, the bilateral HP and A1 showed an intense negative correlation between the contrasts of interest, that is, Tooth Nar and Trauma Rem, as well as, Tooth Rem and Trauma Rem (Tooth Nar and Trauma Rem: Spearman’s rank correlation coefficient [RS] = −0.867 and − 0.617, *P* < 0.01 and *P* < 0.10 for left and right HPs, respectively; RS = −0.983 and − 0.933, *P* < 0.000 and *P* < 0.001 for left and right A1s, respectively. Tooth Rem and Trauma Rem: RS = −0.333 and − 0.517, *P* > 0.10 for both of left and right HPs; RS = −0.483 and − 0.667, *P* > 0.10 and *P* < 0.10 for left and right A1s, respectively) (Supplementary Table S11).

**Table 2:** Coordinates of top negative correlations found in Pt1

Table 2-1**.** Correlation between Tooth Nar and Trauma Rem							
*x*	*y*	*z*	RS	*P*	Anatomy	Side	Label
−44	−36	−10	−0.983	<0.000	MTG/ITG	L	a
−12	−24	−6	−0.983	<0.000	Thalamus/midbrain	L	b
−44	−26	10	−0.983	<0.000	STG	L	c
60	−24	8	−1.000	<0.000	A1/STG	R	d
38	−24	10	−1.000	<0.000	A1/insula	R	e
42	−2	14	−1.000	<0.000	Insula	R	
42	−18	0	−0.983	<0.000	Insula	R	
34	−10	6	−0.983	<0.000	Insula	R	
36	−2	0	−0.983	<0.000	Insula	R	
54	−16	4	−0.983	<0.000	STG	R	
56	12	−22	−1.000	<0.000	STG/pole	R	f
60	2	−20	−0.983	<0.000	MTG	R	g
22	−24	−20	−0.983	<0.000	PH	R	h
16	−12	−6	−0.983	<0.000	Thalamus/HP	R	i
12	−32	−2	−0.983	<0.000	PH	R	
22	32	56	−0.983	<0.000	SFG	R	j
30	−26	58	−0.983	<0.000	CS	R	k
22	−26	62	−0.983	<0.000	CS	R	l
Table 2-2**.** Correlation between Tooth Rem and Trauma Rem							
*x*	*y*	*z*	RS	*P*	Anatomy	Side	Label
−26	−20	−18	−0.983	<0.000	HP	L	m
48	8	−34	−0.983	<0.000	MTG/pole	R	n
18	−16	−18	−0.983	<0.000	PH/HP	R	o
36	−8	2	−0.983	<0.000	Putamen/insula	R	p

Note: Coordinates (*x*, *y*, and *z*) corresponding to the MNI space. Those with RS ≤ −0.983 and ≥6 mm apart from each other are reported. RS, coefficient of Spearman rank correlation at the coordinate voxel; *P*, *P* value for testing no correlation; L, left; R, right; MTG, middle temporal gyrus; pole, temporal pole; CS, central sulcus.

**Figure 7:**
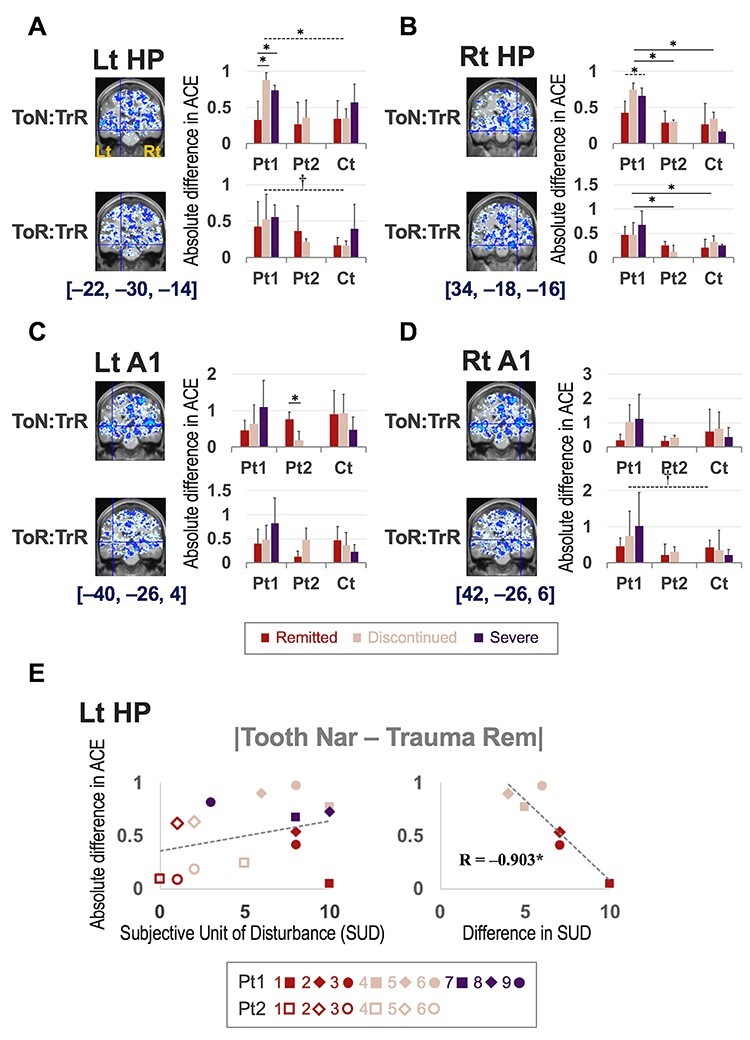
Analyses of differences in ACE between Tooth Nar/Rem and Trauma Rem. Analyses for the ROIs (*A*) in the Lt HP, (*B*) Rt HP, (*C*) Lt A1, and (*D*) Rt A1. Coronal sections show negative correlations between contrast conditions indicated in the leftmost column. ToN, Tooth Nar; TrR, Trauma Rem; ToR, Tooth Rem. Cyan color indicates voxels whose Spearman’s rank correlation coefficients are ≤− 0.8; blue, ≤− 0.5; and ice blue, ≤− 0.2. Hairline crossings align with the center ROI coordinates indicated below the sections. In the bar graphs on the right side, the wine color indicates remitted patients and matched controls; cream, discontinued patients and matched controls; and purple, severe patients and matched controls. The vertical axis indicates absolute ACE difference between the indicated contrast conditions in the ROI. The solid line and dashed line over the bar graphs indicate statistical significance; the solid line indicates a significant difference in the post hoc test between the indicated pair, whereas the dashed line indicates a significant difference as a whole but not in the post hoc test. *, *P* < 0.05; †, *P* < 0.10. Details regarding the statistical analysis can be found in Supplementary Table S12. (*E*) Scatter graphs for absolute differences in ACE between Tooth Nar and Trauma Rem in the Lt HP with horizontal axis of score of SUD (left panel), or difference between the initial SUD score and the last score after EMDR (right panel) (see Supplementary Table S2 for raw data). Plots represent patients as indicated in the bottom part; the numbers 1–9 correspond to IDs in Supplementary Tables S2. The equation indicates Pearson’s product–moment correlation coefficient between values of the vertical and horizontal axes.

We closely examined these four ROIs and found that the magnitude of the discrepancy between the Tooth Nar/Rem and the Trauma Rem was smaller in the remitted group at Pt1 in general (Fig. 7*A*–*D*; Supplementary Table S12). We also found that the discrepancy was greater in Pt1 than in Pt2 and Ct in general (F(2, 189) = 14.042, *P* < 0.000 for one-way ANOVA combining left and right HPs and A1s as well as combining Tooth Nar/Rem and Trauma Rem; Pt1 > Pt2 and Pt1 > Ct by post hoc test at alpha = 0.05) (Fig. 7*A*–*D*). In addition, the left HP ROI demonstrated that the magnitude of the discrepancy between Tooth Nar and Trauma Rem was negatively correlated to SUD changes between the values before and after EMDR (*R* = −0.903, *P* < 0.05), indicating that patients with the smaller discrepancy in activity estimates showed better improvement in the subjective disturbance (Fig. 7*E*). See Supplementary Material for more details (Supplementary Figs S6 and S7; Supplementary Table S12).

## Discussion

### Summary of Results and Negative Correlations between Tooth and Trauma Tasks

Originally, the computation of correlations between task conditions was not planned. We followed a legitimate procedure and found that the results from a conventional SPM suggested a patient’s overall dysfunctions in sensory processing (V1) and a combination of effects in the DMN, including a “failure of deactivation” in the DMN (Bluhm et al. 2009; Akiki et al. 2017) during Trauma Nar (Supplementary Fig. S2), and conversely, an increased activation in the mPFC (Supplementary Fig. S2, Supplementary Table S7) and of the lateral parietal cortex (Supplementary Table S7) during Trauma Rem. The latter might be caused by an intense episodic memory retrieval (Rugg and Vilberg 2013; Kim 2016).

Next, a series of ROI analyses failed to detect significant differences among subject groups (Supplementary Table S9). However, in the course of the ROI analyses, we noticed a strong negative correlation in the bilateral HPs during Tooth Nar with the hyperarousal subscale of IES-R-J (Fig. 4). Interestingly, the same subscale was positively correlated with contrast estimates during Trauma Rem (Fig. 4). By observing this discrepancy, we deduced that the contrast estimates of Trauma Rem might be negatively correlated with those of Tooth Nar in Pt1.

The negative correlation in Pt1 was indeed revealed by the ROI-based correlation matrix (Fig. 5*A*) and by the voxel-based correlation map (Fig. 5*F*,*H*). Both suggested that Pt1 shifted to a separation into daily and traumatic memory modes; Ct had a stable functioning, specifically in remembering; Pt2 was in a transitional phase. The negative correlation in Pt1 specifically occurred in temporal structures including the A1 (Table 2; Fig. 6). It was consistent with the fact that the temporal lobe has relevance to memory functions: The episodic memory of the trauma would involve medial temporal lobes, including the HP and PH, whereas the semantic memory, which affected the patients’ whole life, would involve anterior and inferior temporal lobes (Matthews 2015). The A1 involvement reminds us of a connection between auditory and memory functions; the hearing ability could play a role in the intact memory, cognitive, and/or affective functions in the elderly, brain-injured, and children (Kleim and Jones 2008; Fulton et al. 2015; Simon et al. 2020) whose process might share a mechanism with altered auditory and related systems by stress (Dagnino-Subiabre 2013; Jafari et al. 2017; Pérez-Valenzuela et al. 2019).

We specifically remark the extensive negative correlation near the HP. Importantly, this could not be discovered without our new correlation computation method. The HP is a center of memory consolidation (Liberzon and Sripada 2008) and is involved in altered cognition in PTSD (Fenster et al. 2018). Studies have revealed structural and functional alterations in the HP in PTSD (Bremner et al. 2003, 2008; Vermetten et al. 2003; Geuze et al. 2005; Dickie et al. 2011; Mary et al. 2020). However, the involvement of the HP in PTSD was not consistently indicated (Shin et al. 2006; Akiki et al. 2017; Malejko et al. 2017). For example, activity in the HP was found to be augmented in patients in studies by Osuch et al. (2001) and Piefke et al. (2007), but reduced (Bremner et al. 1999) or augmented after an intervention (Pagani et al. 2007; Peres et al. 2007) in other studies; yet, other studies did not specifically report on HP involvement (Supplementary Table S1). This variation might not only be due to the differences in patient and/or control characteristics, tasks, and methodologies but also due to the negative correlations between the tasks that might have canceled out the differences (Fig. 1). As a result, important clinical symptoms of this disease, that is, negative correlation of activities in the HP between daily and traumatic memories in PTSD, might have been overlooked.

An inter-task correlation method has previously been applied in the context of schizophrenia (Michael et al. 2009), endorsing the usefulness of correlation computation between task conditions. Michael et al. (2009) computed correlations between all possible pairs of voxels and examined the resulting histogram, whereas we took a more straightforward method to directly examine the correlations between tasks. At least for daily and traumatic memories in PTSD, the simple correlation computation employed in our study using neutral and traumatic scripts would be advantageous because it directly elucidates the switching of suppression and enhancement of the patient brain activity.

### Two Alternating Function Modes

The negative correlations in Pt1 revealed the existence of both a daily mode and traumatic memory mode in the patients’ brains. It clearly demonstrated that the patients responded differently to the two scripts. By contrast, positive correlations implied that an individual who intensely activated a brain location in a task also intensely activated the same location in another task. This sounds natural and indicates that the individuals responded similarly to different tasks.

The negative correlation implied that the more the brain activity was suppressed during the Tooth task, the greater the brain activity during the Trauma task and vice versa. Patient type (Supplementary Table S2-2, rightmost column) did not explain the response direction; for example, a severe patient had a low value for Tooth Nar but a high value for Trauma Rem, while another severe patient showed the opposite pattern (Fig. 4). Although preliminary, our findings could respond to the following hypothesis. The HP might be suppressed in patients who severely suffer from a hyperarousal to traumatic memory during daily recognition. To attend to the Trauma task, the patients had to release the suppression to intentionally remember the trauma. It is easy to imagine that this release would make the HP run out of control and increase the contrast estimates. By contrast, patients who maintained an increased arousal level to the trauma in daily recognition might have an active functioning of the HP in peacetime. However, this type might have to suppress or “shut down” the HP during Trauma Rem to protect themselves from the harmful memory. These reverse responses in patients might explain the lack of significant differences between the subject groups in the ANOVA for ROIs (Supplementary Table S9), and also, as discussed earlier, the heterogeneity of the functional imaging results reported thus far (Shin et al. 2006; Akiki et al. 2017; Malejko et al. 2017; Supplementary Table S1). As reference, we went back to conventional SPM to compare the magnitude between Trauma Rem and Tooth Nar and found an increased activity near the HP during Trauma Rem in Pt1 (Supplementary Fig. S8), but the extent was severely limited compared with that of the negative correlation (Fig. 6).

One might consider that a traumatic event would only create a traumatic memory mode in addition to the original intact daily mode. However, our findings clearly indicate that the patients’ neural responses to daily events were no longer unaffected. As discussed above, patients would experience hyperarousal or suppression even during the recognition of a daily activity. We could identify the impact of trauma to split the patients’ mind into two: a traumatic memory mode and a daily mode altered in its nature by trauma.

### Window of Tolerance

Of clinical interest was that the magnitude of changes in contrast estimates between Tooth and Trauma tasks appeared to predict a patient’s prognosis. The absolute difference between Tooth Nar/Rem and Trauma Rem at first scan (Pt1) was smaller in patients who remitted than in others (Fig. 7*A*–*D*; Supplementary Fig. S6; Supplementary Table S12). In addition, in the left HP, we found that the absolute difference of activities in Pt1 had a negative correlation with the SUD improvement (Fig. 7*E*); the smaller the difference in activities before EMDR, the greater the reduction in subjective disturbance by EMDR. In fact, the discrepancy between Tooth Nar/Rem and Trauma Rem appeared to reflect the severity of the symptoms. First, the discrepancy increased successively in remitted, discontinued, and severe in Pt1 in general (the second to last line of Supplementary Table S12-2). Next, the absolute discrepancy was greater in Pt1 than in Pt2 and Ct (the last line of both Supplementary Table S12-1 and S12-2).

These findings appear to be consistent with the “window of tolerance” model (Ogden and Minton 2000; Corrigan et al. 2011), which refers to an optimum zone of arousal where patients can efficiently function. Patients with PTSD often experience two extremes: hyperarousal (e.g., anger) or hypoarousal (e.g., dissociation) (Corrigan et al. 2011). The two extremes frequently switch and prevent patients from processing information properly; we can think, experience, and function properly only within the “optimum arousal zone” in between the two extremes (Ogden and Minton 2000; Corrigan et al. 2011). The greater the discrepancy between the extremes, the more severe the pathology. Consequently, patients might be driven outside the optimal window during not only traumatic remembrance but also during daily recognition; both situations would hamper proper information processing, involuntarily leading to hyperarousal or dissociation. The EMDR treatments apparently reduced this discrepancy (Fig. 7) in addition to the scores of the psychological assessment subscales (Supplementary Table S6), bringing patients back to the optimal window range. The negative correlations in contrast estimates between the Tooth and Trauma tasks were greatly reduced in Pt2 (Fig. 7; Supplementary Table S12), suggesting that the patients regained their capability to process information properly for both daily recognition and remembering traumatic memories. In fact, the coordination of patients’ arousal levels within their tolerance is key in the EMDR treatment (Paulsen and Lanius 2009). We thus claim that EMDR helped our patients to resolve their divided mind.

### Limitations

The relatively small size of sample may be a source of bias. Further, we included patients taking medication that might affect brain activities; we chose a procedure with no drug washout to minimize the effect of the study on the treatment process. Because we aimed to observe general activity behaviors using a small sample, we did not avoid multiple comparisons as well as circular analyses.

## Conclusions

By combining fMRI with a script-driven imagery task, we discovered a negative correlation of activity estimates between daily recognition and trauma memory remembrance in patients with PTSD. Its clinical impact was observed in the reduction of the magnitude of the discrepancy between the two conditions in remitting patients after EMDR treatment. We thus propose the application of a correlation analysis to examine patients with PTSD, who often present the extremes of hyper- and hypoarousals. Further research is necessary to identify the contribution of correlation analysis between different conditions during task-employed fMRI not only to clinical neuroimaging but also to cognitive neuroscience.

## Notes

We would like to express our warmest gratitude to all participants who attended this painstaking experiment. We are thankful for the understanding of attending psychiatrists and clinical psychologists in charge of the therapies, specifically Ms Aina Iio and Ms Tomoyo Isobe. We would also like to thank the MRI technicians, specifically Mr Naoki Ooishi, Mr Masanori Kawate, and Mr Youhei Yokoyama, and other people who supported our research whom we do not specifically mention here. *Conflict of Interest*: Y.T. is an endowed chair of the Nagoya University Graduate School of Medicine and is supported financially by the private company HIMEDIC, Nagoya, Japan. The other authors have no conflicts of interest to declare.

## Funding

Challenging Exploratory Research of the Japan Society for the Promotion of Science (JSPS) (KAKENHI #15K13140 to J.I.).

## Supplementary Material

SupplementaryMaterials_ptsd_210312_bk_tgab021
