# Supplementary material for: How Memory Switches Brain Responses of Patients with Post-traumatic Stress Disorder
Source: Cereb Cortex Commun. 2021 Mar 20;2(2):tgab021. doi: 10.1093/texcom/tgab021 (PMC8176146; doi:10.1093/texcom/tgab021)
Supplement: SupplementaryMaterials_ptsd_210312_bk_tgab021 [file supplementarymaterials_ptsd_210312_bk_tgab021.zip › SupplementaryMaterials_ptsd_210312_bk_tgab021.pdf]

Supplementary Materials

for

How memory switches brain responses of patients with posttraumatic stress disorder

Authors

Jun Inoue, Kayako Matsuo, Toshiki Iwabuchi, Yasuo Takehara, Hidenori Yamasue

Contents

Summary of script-driven imagery task studies (**Supplementary Table S1**) …… page S3
Patient descriptions (**Supplementary Tables S2, S3, and S4**) …… page S6
fMRI paradigm (**Supplementary Figure S1**) …… page S12
Physiological data analysis (**Supplementary Table S5**) …… page S14
Assessment score analysis (**Supplementary Table S6**) …… page S16
Brief explanation of the EMDR procedure …… page S18
Region-of-interest (ROI) analyses: overview …… page S19
Activation maps by standard SPM analysis …… page S21
(**Supplementary Figure S2, Supplementary Table S7**)
Details of ROIs (**Supplementary Table S8**) …… page S23
Correlations between assessment scores and ROIs …… page S24
(**Supplementary Figures S3 and S4**)
ANOVA of activity estimates in ROIs (**Supplementary Table S9**) …… page S28
Analysis of correlation matrices …… page S31
(**Supplementary Figure S5, Supplementary Table S10**)
Correlations in ROIs between Tooth Nar/Rem and Trauma Rem …… page S34
(**Supplementary Table S11**)
Analysis of discrepancies between Tooth Nar/Rem and Trauma Rem …… page S35
(**Supplementary Figures S6 and S7, Supplementary Table S12**)
SPM comparison between Trauma Rem and Tooth Nar …… page S40
(**Supplementary Figure S8**)
References …… page S41

Summary of script-driven imagery task studies

Supplementary Table S1 shows a summary of the functional brain imaging studies that used a script-driven imagery task. This table was based on our own literature search and several review or meta-analysis papers (Lanius et al. 2006; Thomaes et al. 2014; Malejko et al. 2017; Thome et al. 2020).

Supplementary Table S1. Functional brain imaging studies using a script-driven imagery task

Study	Patients (N) *1	Trauma	Controls (N) *2	Intervention *3	Scripts *4	Method, special note *5	Main neuroimaging results *6	Assessment and scale *7
Rauch et al. (1996)	PTSD (8)	Various	None	None	TR and N	PET	(Inc) right LM, VC; (Dec) left IFG, MTC	CAPS=69.6, DES=17.1, IES-R=74.0, M-PTSD=124.8
Levin et al. (1999)	PTSD (6)	Various	None	EMDR	TR and N	SPECT, Pre-Post	(Post>Pre) ACC, left FL	CAPS: Pre=62, post=31, Davidson: Pre=57, Post=30, IES: Pre=33, Post=13, HDS; Pre=5, Post=6
Bremner et al. (1999)	PTSD (10)	Childhood sexual abuse	TEC (12)	None	TR and N	PET	(Inc) PCC, MC; (Dec) mPFC, HP, VC	The Early Trauma Inventory=948, PTSD symptom Scale=19, CADSS=2.3
Shin et al. (1999)	PTSD (8)	Childhood sexual abuse	TEC (8)	None	TR and N	PET	(Inc) OFC, anterior TP; (Dec) ACC	CAPS=64.8, BDI=15.6, STAI-Trait=50.6, STAI-State=38.1
Lanius et al. (2001)	PTSD (9)	Various	TEC (9)	None	TR and N	fMRI	(Dec) ACC, mPFC, thalamus	CAPS=75
Osuch et al. (2001)	PTSD (11)	Various	None	None	TR	PET, flashback	(Inc with flashback) BS, left HP, SS, CBL; (Dec with flashback) DLPFC, right MTC	CAPS intrusion=3.7, avoidance=5.8, hyperarousal=4.6
Lanius et al. (2002)	PTSD (7, dissociative)	Childhood sexual/physical abuse	TEC (10, dissociative)	None	TR and N	fMRI	(Inc) PFC, LM	CAPS=87, DES=43,
Lanius et al. (2003)	PTSD (10)	Various	TEC (10)	None	TR, TUR sad, TUR anxious, and N	fMRI	(Dec) ACC, thalamus	CAPS=75
Driessen et al. (2004)	PTSD+BPD (6)	Various	TEC (6, BPD)	None	TR and N	fMRI	(Inc) right anterior TL, amygdala, PCC, OL, CBL	CTQ=80.4, IES-R=77.2, DES=18.1,BDI=25.8, SCL-90-R=99.3
Shin et al. (2004)	PTSD (17)	Vietnam war veterans (7 male combat veterans, 10 female nurse veterans)	TEC (19)	None	TR and N	PET, neg corr	(Inc) left amygdala; (Dec) mPFC	male, female; CAPS=80.3,69.5, SCL-90-R depresssion subscale score=1.4, 1.9
Britton et al. (2005)	PTSD (16)	Combat	TEC (15), HC (14)	None	TR, TUR highly stressful, and N	PET	(Inc) vmPFC; (Dec) rostral ACC	CAPS=61.3, IES=68.4, DES=21.7, TAS=71.9, BDI=21.3, STAI-State=45.6, STAI-Trait=49.5
Lanius et al. (2005)	PTSD (21: dissociative=10, flashback =11)	Various	TEC (10)	None	TR and N	fMRI, FC	(Dec FC for dissociative) VLT and PH; (Inc FC for dissociative) VLT and IS; (Inc FC for flashback) ACC and IFG	dissociative, flashback; CAPS=90, 72, DES=34.8, 17.9

Pagni et al. (2007)	PTSD (15: R=11, NR=4)	Job related (train drivers)	TEC (27)	EMDR	TR	SPECT, Pre-Post	(R>NR) HP; (Inc) OFC, TP	None
Peres et al. (2007)	PTSD (16, subthreshold)	Various	WL (11, subthreshold PTSD)	CBT	TR	SPECT, Pre-Post, Pos corr	(Post>Pre) parietal lobe, left HP, thalamus, left PFC	CAPS: Pre=45, Post=20, IES: Pre=35, Post=6, BDI: Pre=14, Post=4, BAI: Pre=32, Post=10
Lanius et al. (2007)	PTSD (26: PTSD=11, PTSD+MDD=15)	A motor vehicle accident	TEC (16)	None	TR and N	fMRI	(Dec) ACC, right ventrolateral PFC; (Inc in PTSD-MDD) left insula; (Inc in PTSD+MDD) ACC, PCC	PTSD, PTSD+Depression: CAPS=56.4, 76.0, BDI=18.7, 37.5
Piefke et al. (2007)	PTSD (6, acute)	A severe accident	None	None	TR, TUR negative, and N	fMRI	(Inc) amygdala, HP, lateral TL, retrosplenial, ACC	IES=19.2, HADS Anxiety=7.5, HADS Depression=5.2
Hopper et al. (2007b)	PTSD (27)	Various	None	None	TR and N	fMRI, RSDI scale	(Inc with Ree) right anterior IS; (Dec with Ree) right rostral ACC; (Dec with Avo) rostral ACC; (Inc with Dis) left mPFC, right STC; (Dec with Dis) left STC	CAPS=69, BDI=30.9, BAI=21.4, DES=9.3, PDEQ=29.5
Frewen et al. (2008)	PTSD (26)	A motor vehicle accident	None	None	TR and N	fMRI	(Inc) right posterior IS, PCC; (Dec) ACC, vmPFC, anterior IS, right IFG	CAPS=80.38, TAS-20=57.77
Lindauer et al. (2008)	PTSD (20: R=10, WL=10)	Various	TEC (15)	BEP	TR	SPECT, Pre-Post	(Inc) right IS, right S/MFG; (Dec in R) right MFG; (Inc in R) left STC, S/MFG	SI-PTSD: Pre=11.7, Post=3.7, STAI: Pre=58, Post=45.7
Ludäscher et al. (2010)	PTSD (10, PTSD+BPD)	Various	TEC(5, BPD)	None	dissociation-inducing and N	fMRI	(Inc) left ACC; (Inc with Dis) IS; (Dec with Dis) right PH	FDS(trait dissociation score)=28.8, DSS-acute(State dissociation score)=1.1, BSL=2.3
Peres et al. (2011)	PTSD (12, partial)	Job related (police)	WL (12, partial PTSD), TEC (12)	Inpatient CBT group	TR, pleasant, and N	fMRI, Pre-Post	(Inc) mPFC; (Dec) amygdala	CAPS: Pre=48, Post=19, IES: Pre=38, Post=10, BDI: Pre=13,Post=7, BAI: Pre=28,Post=11
Fani et al. (2011)	PTSD (7)	Various	PTSD (6, placebo)	Paroxetine	TR and N	PET, RCT, Pre-Post	(Inc for all) ACC; (Inc for paraxetine) OFC	CAPS: Pre=84.9 , Post=26.3
Mickleborough et al. (2011)	PTSD (17)	Various	TEC (26)	None	TR and N	fMRI, pain	(Inc) head of caudate, thalamus; (Dec with DES) right amygdala, left putamen	CAPS=70.1, BDI=10.6, DES=9.3, CADSS=4
Whalley et al. (2013)	PTSD (10)	Various	TEC (15), Depression (14)	None	flashback-related words and phrases	fMRI, flashback	(Inc with flashback) S/MC; (Dec with flashback) midbrain, PH, precuneus/PCC	BDI=27.1, BAI=23.3, PDS=33.5
Dahlgren et al. (2018)	PTSD (12)	Combat	Twin HC (12), TEC (14) and twin HC (14)	None	TR, TUR stressful, and N	fMRI	(Inc) mPFC	CAPS=52, CTQ=39.6, BDI=10, BAI=9.25, MAST=4.58, Combat Severity=8
Inoue et al. (current)	PTSD (9: Pre=9, Post=6)	Various	HC (9)	EMDR	TR and N	fMRI, Pre-Post	(Neg corr between traumatic and daily scripts) HP, TL; (Dec with Hyp) HP	CAPS; Pre＝80.3, Post＝47.2, IES; Pre=52.2, Post=35.8, DES; Pre=3 3 .9, Post=19.0, BDI; Pre=32.3, Post=19.7, STAI-Trait; Pre=67.3, Post=60.7, STAI-State; Pre=66.7, Post=59.5

Notes. *1: BPD, borderline personality disorder; MDD, major depressive disorder; R, responder to intervention; NR, non-responder to intervention; WL, wait-list; Pre, before intervention; Post, after intervention.

*2: TEC, trauma-exposed, healthy controls; HC, healthy controls.

*3: EMDR, eye movement desensitization processing; CBT, cognitive behavioral therapy; BEP, brief eclectic psychotherapy.

*4: TR, trauma-related; N, neutral; TUR, trauma-unrelated.

*5: Pre-Post, before and after intervention; Neg corr, negative correlation between brain areas; FC, functional connectivity; Pos corr, positive correlation between brain areas; RSDI, Responses to Script-Driven Trauma Imagery Scale.

*6: selection arbitrary. Inc, increase; LM, limbic/paralimbic; VC, visual cortex; Dec, decrease; IFG, inferior frontal gyrus; MTG, middle temporal gyrus; ACC, anterior cingulate cortex/gyrus; FL, frontal lobe; PCC, posterior cingulate cortex; MC, motor cortex; mPFC, medial prefrontal cortex; HP, hippocampus; OFC, orbitofrontal cortex; TP, temporal pole; SS, somatosensory; BS, brainstem; CBL, cerebellum; DLPFC, dorsolateral prefrontal cortex; MTC, medial temporal cortex; PFC, prefrontal cortex; TL, temporal lobe; OL, occipital lobe; vmPFC, ventromedial prefrontal cortex; VLT, ventrolateral thalamus; IS, insula; PL, parietal lobe; STC, superior temporal cortex; S/MFG, superior/middle frontal gyrus; PH, parahippocampal cortex; S/MC, sensory and motor cortices; A1, primary auditory area.

*7: CAPS, Clinical Administered PTSD Scale; DES, the Dissociative Experience Scale; IES, the Impact of Event Scale; BAI, the Beck Anxiety Inventory; BDI, Beck Depression Inventory; SI-PTSD, the Structured Interview for Posttraumatic Stress Disorder, M-PTSD, Civilian Version of the Mississippi PTSD scale; Davidson, Davidson Self-Rating PTSD Scale; HDS, Hamilton Depression Scale; CTQ, Childhood Trauma Questionnaire; SCL-90-R, Symptom Check List 90, Revised; MAST, Michigan Alcoholism Screening Test; Combat severity, a combat exposure severity index for Vietnam era veterans; TAS; Toronto Alexithymia Scale; STAI, State-Trait Anxiety Inventory; CADSS, Clinician Administered Dissociative States Scale; PDEQ, Peri traumatic Dissociative Experiences Questionnaire; FDS, Fragebogen zu Dissociative Symptomen; DSS-acute, Dissociation Tension Scale-acute; BSL, the self-administered Borderline Symptom List: HADS, Hospital Anxiety and Depression Scale; PDS, Posttraumatic Diagnostic Scale.

Patient descriptions

The research progressed as follows. When we applied for a three-year research grant from a national society (Japan Society for the Promotion of Science; JSPS), considering the past record of our Department of Psychiatry, we estimated that we could collect data from at most 20 patients with posttraumatic stress disorder (PTSD). During the study period, we actually invited all 11 patients with PTSD who visited to our hospital to participate in the study, but two declined. When we got consent from a patient, we searched for a matched control from our research community and asked him or her to participate. All participant recruitments and the measurements described in this manuscript occurred during the funded study period from April 2015 to March 2018. No further follow-up measurements regarding this study have occurred until now (March 2021). We do not believe that the measurements hampered the patients' treatment. The treatment, when necessary, has normally continued after the end of measurements in the same way as it did for other patients. See **Figure 2** (main text) for the CONSORT diagram (Moher et al. 2010). Individual information has been summarized in **Supplementary Tables S2, S3, and S4**.

Supplementary Table S2. Participant demographics

Supplementary Table S2-1. Patients #1

ID	Index trauma	Gender	Age	Handedness	Education (years)	In/Out 1	SUD 1
1	Transportation accident	F	36	100	14	Out	10
2	Transportation accident	F	35	28.6	16	Out	8
3	Sexual betrayal	F	27	100	16	Out	8
4	Fell into chemicals	F	30	100	16	Out	10
5	Abusive assault by a parent	M	27	100	16	Out	6
6	Transportation accident (victimizer)	M	27	100	14	Out	8
7	Sexual assault	F	50	100	12	In	8
8	Maltreatment, sexual assault	F	34	-100	9	In	10
9	Incest	F	29	100	12	Out	3
	Mean (SD)	–	32.8 (7.0)	69.8 (64.0)	13.9 (2.3)	–	7.9 (2.1)

Supplementary Table S2-2. Patients #2

ID	Times EMDR	Interval (days)	In/Out 2	SUD 2	Type
1	7	314	out	0	Remitted
2	7	356	out	1	Remitted
3	15	477	out	1	Remitted
4	7	406	out	5	Discontinued
5	9	117	out	2	Discontinued
6	7	366	out	2	Discontinued
7	–	–	–	–	Severe
8	–	–	–	–	Severe
9	–	–	–	–	Severe
Mean (SD)	8.7 (2.9)	339.3 (111.4)	–	2.8 (2.8)	–

Supplementary Table S2-3. Matched controls

ID	Gender	Age	Handedness	Education (years)
1	F	30	100	18
2	F	34	100	18
3	F	25	100	18
4	F	32	100	18
5	M	26	100	18
6	M	27	100	18
7	F	51	100	21
8	F	30	100	18
9	F	28	100	18
	Mean (SD)	31.4 (7.4)	100 (0)	18.3 (0.9)

Notes. Each control participant was paired with the same ID number patient. Age: age at the 1st scan. Handedness: Edinburgh Handedness Inventory score. In/Out 1: inpatient or outpatient at the time of the 1st scan. SUD 1: SUD score at the time of the 1st scan. Times EMDR: session number for phases 4 to 7 in EMDR (combined) conducted between the 1st and the 2nd scans. Interval: number of days between the 1st and 2nd scans. In/Out 2: inpatient or outpatient at the time of the 2nd scan. SUD 2: SUD score at the time of the 2nd scan. Type: indication at the end point of each patient (see text for the definition).

Supplementary Table S3. Patient indications and medications

ID	Diagnoses (DSM-5)	Trauma onset age	Psychiatric medications at the time of the first scan	Psychiatric medications at the time of the second scan
1	PTSD	30	Escitalopram 10 mg, Olanzapine 2 mg	Escitalopram 20 mg, Asenapine 5 mg, Clotiazepam 5 mg
2	PTSD	34	Paroxetine 12.5 mg	–
3	PTSD, Graves' disease	25	Escitalopram 20 mg	Escitalopram 20 mg, Trazodone 25 mg
4	PTSD	28	–	–
5	PTSD, neurodevelopmental syndromes	14	Duloxetine 60 mg, Etizolam 0.5 mg, Atomoxetine 120 mg, Eperisone 100 mg, Zolpidem 10 mg, Aripiprazole 6 mg	Duloxetine 60 mg, Etizolam 1.5 mg, Eperisone 100 mg, Atomoxetine 120 mg, Brotizolam 0.25 mg, Prazosin 1 mg
6	PTSD, depression	26	Sertraline 100 mg, Olanzapine 5 mg, Flunitrazepam 2 mg, Mirtazapine 30 mg	Sertraline 100 mg, Olanzapine 5 mg, Flunitrazepam 2 mg, Mirtazapine 30 mg
7	PTSD, DID	sexual assault 9, 27	–	–
8	PTSD, DID	maltreatment 3, sexual assault 25	Fluvoxamine 100 mg, Etizolam 1.5 mg, Flunitrazepam 2 mg, Zolpidem 10 mg	–
9	PTSD, DID	12	Keishikashakuyakuto 7.5 g, Shimotsuto 7.5 g	–

Notes. ID numbers correspond to those in **Supplementary Table S2**. DID: dissociative identity disorder.

Supplementary Table S4. Psychological assessment scores

Supplementary Table S4-1. CAPS

ID	Pt1				Pt2			
	Ree	Avo & Num	Hyp	Total	Ree	Avo & Num	Hyp	Total
1	22	14	15	51	5	0	8	13
2	14	31	26	71	7	20	18	45
3	28	37	27	92	19	35	23	77
4	30	22	15	67	10	7	9	26
5	21	49	31	101	8	39	29	76
6	32	37	31	100	6	27	13	46
7	11	35	12	58	–	–	–	–
8	33	41	32	106	–	–	–	–
9	29	35	17	81	–	–	–	–
Mean	24.4	33.4	22.9	80.8	9.2	21.3	16.7	47.2
SD	7.9	10.3	8.1	20.1	5.1	15.4	8.3	25.9
Mean for upper 6	24.5	31.7	24.2	80.3	9.2	21.3	16.7	47.2
SD	6.7	12.4	7.4	20.4	5.1	15.4	8.3	25.9

Supplementary Table S4-2. IES-R-J

ID	Pt1				Pt2			
	Int	Avo	Hyp	Total	Int	Avo	Hyp	Total
1	11	7	8	26	2	1	5	8
2	15	17	18	50	6	12	12	30
3	24	15	19	58	16	23	13	52
4	24	15	14	53	9	21	10	40
5	28	14	24	66	15	14	20	49
6	27	14	19	60	11	19	6	36
7	22	19	15	56	–	–	–	–
8	29	25	21	75	–	–	–	–
9	25	24	12	61	–	–	–	–
Mean	22.8	16.7	16.7	56.1	9.8	15.0	11.0	35.8
SD	6.0	5.5	4.9	13.5	5.3	8.0	5.4	15.9
Mean for upper 6	21.5	13.7	17.0	52.2	9.8	15.0	11.0	35.8
SD	6.9	3.4	5.4	14.0	5.3	8.0	5.4	15.9

Supplementary Table S4-3. DES-II

ID	Pt1	Pt2
1	290	380
2	850	540
3	210	250
4	470	200
5	1430	1390
6	700	440
7	1690	–
8	1650	–
9	1260	–
Mean	950.0	533.3
SD	575.6	437.6
Mean for upper 6	658.3	533.3
SD	448.6	437.6

Supplementary Table S4-4. RSDI

	Pt1				Pt2				Ct			
ID	Ree	Avo	Dis	Total	Ree	Avo	Dis	Total	Ree	Avo	Dis	Total
1	12	13	5	30	6	0	0	6	0	0	0	0
2	19	6	15	40	9	2	2	13	7	0	0	7
3	22	11	0	33	8	2	0	10	0	0	0	0
4	24	10	1	35	16	2	0	18	7	2	0	9
5	8	4	6	18	8	4	6	18	15	4	5	24
6	22	13	14	49	21	15	5	41	7	0	0	7
7	21	2	0	23	–	–	–	–	7	5	2	14
8	0	18	12	30	–	–	–	–	2	0	0	2
9	20	0	0	20	–	–	–	–	4	0	0	4
Mean	16.4	8.6	5.9	30.9	11.3	4.2	2.2	17.7	5.4	1.2	0.8	7.4
SD	8.1	5.9	6.3	9.9	5.9	5.5	2.7	12.3	4.7	2.0	1.7	7.7
Mean for upper 6	17.8	9.5	6.8	34.2	11.3	4.2	2.2	17.7	6.0	1.0	0.8	7.8
SD	6.4	3.7	6.4	10.3	5.9	5.5	2.7	12.3	5.6	1.7	2.0	8.8

Notes. ID numbers correspond to those in **Supplementary Table S2**. Pt1: at the time of patients' first scan. Pt2: at the time of patients' second scan. Ct: matched controls. Ree: re-experiencing. Avo & Num: avoidance and numbing. Hyp: hyperarousal. Int: intrusion. Avo: avoidance. Dis: dissociation. SD: standard deviation.

Regarding EMDR application with regard to **Supplementary Table S2**, we add the following information.

- Three certified EMDR therapists were involved in this study.
- ID1 to 6: EMDR treatment through Phases 1 to 8 involved 12 to 25 sessions. The column "Times EMDR" in **Supplementary Table S2** shows the number of sessions from Phases 4 to 7.
- ID2 to 3: Although the SUD for the main trauma was 1 instead of 0 in Phase 4, the therapist evaluated it to be "ecologically" valid to proceed to Phase 5 according to the manual (Shapiro 1995, 2001). The manual suggests not to proceed to Phase 5 until the SUD comes down to 0 in a normal condition unless an "ecologically" valid reason could explain the SUD 1 (e.g., a prolonged trial continued).
- ID4: Although the SUD for the main trauma evaluated in Phase 4 once came down to 0, the symptoms relapsed during the treatment thereafter and the SUD increased to 5 at the end of the treatment.
- ID7 to 9: Although we were able to introduce these patients up to Phase 2, we decided not to proceed to Phase 3 due to their severe dissociation (International Society for the Study of Trauma and Dissociation 2011).

fMRI paradigm

We provide additional explanation on our adapted fMRI paradigm from a previously established script-driven imagery task (Rauch et al. 1996; Lanius et al. 2002, 2005), to which we made minor modifications as follows. First, we presented the text of the narration on the display monitor in addition to the narration itself via the headphones; the original paradigm presented the material orally but not visually. This modification was necessary to address the loud noise from the scanner, which might affect the ability of the participants to listen to the narration; a secure presentation of the scripts would work to lower patient discomfort during the experiment. Next, we explicitly divided the period after the stimulation (i.e., "narration" plus "remembering") into "breathing" and "fixation." This division was to minimize the effect by head movements during "breathing," which might become agitated after the narration; we specified only the "fixation" blocks as baseline in the contrast estimation by disregarding the "breathing" blocks. **Supplementary Figure S1** illustrates the paradigm's time course.

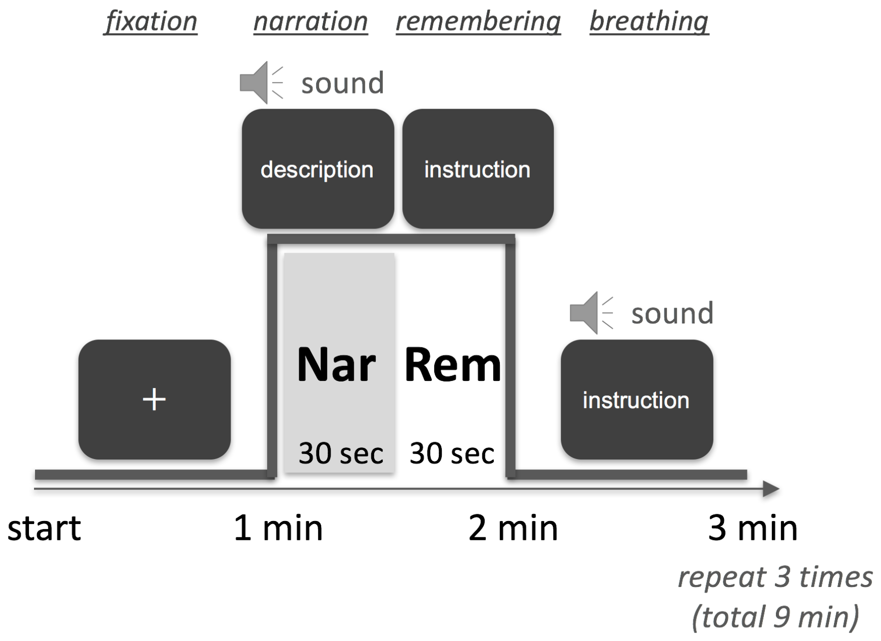

Supplementary Figure S1. fMRI paradigm. A visual stimulus was displayed in all blocks ("fixation," "narration," "remembering," and "breathing"). Blocks "narration" and "breathing" also presented the same text on the monitor that was presented orally. Nar: "narration" contrasted to "fixation." Rem: "remembering" contrasted to "fixation."

The paradigm was developed specifically by referring to the study by Rauch et al. (1996). We chose tooth brushing for the contrast task because it is neutral and seemingly unrelated to almost any kinds of trauma. We controlled the following points between the Tooth

and Trauma scripts as much as possible:
(1) Both Tooth and Trauma scripts had a temporal sequence of the subject's own experiences.
(2) We tried our best to include personalized actions and/or feelings in both scripts, although this depended on the traumatic event of each patient.
(3) To control the amount of auditory stimulation, we matched the average number of mora (i.e., the linguistic unit of time in Japanese) in the Trauma narrations across patients to that in the Tooth narration (see Materials and Methods, fMRI paradigm).

Here we present examples of English translations of narration scripts (originally in Japanese).

Script for the Tooth task (common for all participants):
Remember the following experience of your own. I am going to brush my teeth. I pick up my toothbrush and put some toothpaste on it. Then, I place it on my teeth and move it back and forth and side to side to clean my teeth in sequence; front teeth, back teeth, outside, and inside, and when all these are cleaned, I put some water in a cup, put it into the mouth, rinse, and spit it out. Repeat the rinsing twice or thrice until all toothpaste is gone from my mouth. I feel fresh and clean in my mouth.

An example of traumatic script (for patient ID2 and her counterpart control):
Remember the following experience of yours. One morning, I was driving my daughter and son to school. As the light turned yellow, I put my foot to the brake. Just at that moment, a car came into my sight diagonally from behind, there was a sound “bang”, and a shock ran through my body. My car slid across with a “zzz” sound and stopped. I was stunned and frozen for a while. Then, I jumped to look at my children. When my daughter and I caught each other’s eyes, she burst into tears.

Physiological data analysis

We analyzed the physiological data (pulsation and respiration) acquired during the fMRI sessions. We originally expected to observe a lower heart rate in patients with high dissociation during the Trauma task (Sack et al. 2012).

Analysis methods

The number of peaks in pulse and breathing during fMRI runs was counted using MATLAB functions (The MathWorks, Inc. MA, USA). A patient (Pt1) lacked the data during the Tooth task because of a machine malfunction. We defined counting thresholds by data behaviors as follows. For pulse, we counted peaks above the minimum height at one third of the maximum and with the minimum prominence (depth between one and the next peak) of also one third of the maximum. For breathing, we counted peaks above the minimum height at half of the maximum plus minimum and with the minimum prominence of one tenth of the maximum minus minimum.

Using these counts, we conducted a one-way analysis of variance (ANOVA) to explore differences by group (Pt1, Pt2, and Ct). We also computed Pearson's correlation coefficients between counts and the subscales of neuropsychological assessments obtained.

Results

ANOVA indicated no significant differences between groups. We found significant correlations between physiological data and RSDI subscales (**Supplementary Table S5**). In general, pulse and breathing counts increased according to the increase of the subscale scores in the Trauma task (i.e., positive correlations). Negative correlations were found in Ct1 between the pulse and the avoidance subscale of RSDI; as the avoidance score increased, the pulse became slower in control participants. We did not find any tendencies specific to patients with dissociative identity disorder (IDs 7 to 9 in **Supplementary Tables S2, S3, and S4**) in these analyses.

Discussion

Two findings were noted. One was that the significant correlations were only found in subscales of RSDI. The RSDI was to evaluate emotional responses evoked during the Trauma task (Hopper et al. 2007a, 2007b). Thus, it appeared to indicate the success in the performance of our experiments by sensitively detecting the physiological changes associated with the feelings evoked during the Trauma script. If this was the case, the correlations found during the Tooth task (at avoidance and dissociation of RSDI) might reflect an intrinsic physiological tendency of participants to some extent.

The other finding noted was the significant correlations (including $p<0.10$) found in

Pt2, but not in Pt1, during the Trauma task (**Supplementary Table S5**, re-experiencing and avoidance of RSDI). It might suggest improved self-evaluation ability in Pt2, which might be enhanced during the EMDR treatment, possibly even to a better level than that in control participants.

Supplementary Table S5. Significant correlations found between physiological data and assessment scores

	RSDI			IES-R-J
	Re-experiencing	Avoidance	Dissociation	Hyperarousal
Tooth Puls	–	Ct*(neg)	–	Pt†
Trauma Puls	–	Ct*(neg)	–	Pt†
Puls (Tr – To)	All**, Pt*, Pt2†	–	–	–
Tooth Resp	–	All*	All*, Pt†	–
Trauma Resp	All*	All**, Pt†, Pt2†	–	–
Resp (Tr – To)	All*, Pt†, Pt2†	All†, Pt2*	–	–

Notes. Tooth or Trauma: data during Tooth task or Trauma task, respectively. Puls: pulse data. Resp: respiration (breathing) data. Tr – To: difference between Trauma and Tooth. All: all data available in patients and controls. Pt: patients' data available including both the first and second scans. Pt2: data by patients' second scans. Ct: data by controls. **: p<0.01. *: p<0.05. †: p<0.10. Only Ct*(neg) had a negative correlation, while others had positive correlations.

Assessment score analysis

We investigated a symptomatic improvement with EMDR by conducting a paired t-test using the assessment subscale scores (**Supplementary Table S4**) of six patients who attended both the first and the second scans. Most of the subscales demonstrated an improvement after EMDR (i.e., reduction of the score values) as shown in **Supplementary Table S6**. We also analyzed the subscale scores of RSDI including controls. Effect size and the 95% confidence interval were computed by referring to a practical guide (Nakagawa and Cuthill 2007). We also conducted a repeated-measures ANOVA (within factors of Group 3 and Subscale 3) for the subscale scores of RSDI using six matched subjects. We ignored the multiple comparisons problem to observe the overall tendency. The results suggested that the patients, who were more sensitive to the trauma script than controls, became similar to controls after EMDR.

Supplementary Table S6. Summary of assessment score analysis

Supplementary Table S6-1. Paired t-tests for assessment subscale scores of symptoms

		t-value	p-value	Hodges' g	95% confidence interval
CAPS	Re-experiencing	5.277	0.003**	2.561	0.714 – 4.408
	Avoidance & Numbing	5.515	0.003**	0.739	0.241 – 1.237
	Hyperarousal	3.294	0.022*	0.957	0.138 – 1.776
	**Total**	**5.842**	**0.002****	**1.425**	**0.436 – 2.414**
IES-R-J	Intrusion	8.296	0.000***	1.892	0.651 – 3.133
	Avoidance	-0.552	0.605	-0.216	-0.803 – 0.371
	Hyperarousal	4.045	0.010*	1.103	0.235 – 1.970
	**Total**	**6.434**	**0.001****	**1.092**	**0.344 – 1.840**
DES-II		1.744	0.142	0.282	-0.079 – 0.644

Notes. N=6. ***: p<0.001. **: p<0.01. *: p<0.05. Mean and standard deviation of scores in **Table 1** (main text) as well as in **Supplementary Table S4**. Hodges' g was computed using formulae #1 and #2 whereas the 95% confidence interval was computed using formulae #15 and #18 in the study by Nakagawa and Cuthill (2007).

Supplementary Table S6-2. Paired t-test for RSDI scores

	t-value	p-value	Hodges' g	95% confidence interval
Pt1 and Pt2 (6 people each, df=5)				
Re-experiencing	2.972	0.031*	1.060	0.102 – 2.017
Avoidance	2.286	0.071	1.141	-0.056 – 2.339
Dissociation	2.120	0.087	0.953	-0.012 – 1.918
**Total**	**3.841**	**0.012***	**1.449**	**0.291 – 2.608**
Pt1 and Ct (9 people each, df=8)				
Re-experiencing	3.523	0.008**	2.112	0.721 – 3.503
Avoidance	3.081	0.015*	2.101	0.568 – 3.635
Dissociation	2.283	0.052	1.406	0.206 – 2.605
**Total**	**4.785**	**0.001****	**3.351**	**1.380 – 5.322**
Pt2 and Ct (6 people each, df=5)				
Re-experiencing	1.814	0.129	0.932	-0.229 – 2.093
Avoidance	1.319	0.244	0.785	-0.503 – 2.072
Dissociation	1.661	0.158	0.555	-0.161 – 1.271
**Total**	**1.832**	**0.127**	**0.918**	**-0.209 – 2.045**

Same notes as in **Supplementary Table S6-1**.

Supplementary Table S6-3. Repeated-measures ANOVA for RSDI scores

Variable	F	df	p-value	G.O.^2	Post hoc test
Group	11.336	2,10	0.003**	0.344	Pt1>Ct, Pt1>Pt2, Pt2=Ct
Subscale	18.935	2, 10	0.000***	0.358	Ree>Avo, Ree>Dis, Avo=Dis
Interaction	0.985	4, 20	0.438	-0.001	–

Notes. N=12 (Pt1=Pt2=6, Ct=6). ***: p<0.001. **: p<0.01. G.O.^2: generalized omega squared. Holm's sequentially rejective Bonferroni procedure was used for post hoc test. Ree: re-experiencing. Avo: avoidance. Dis: dissociation.

Brief explanation of the EMDR procedure

We adhered to the standard protocol of EMDR (Shapiro 1995, 2001). The protocol included the following eight phases (Shapiro 2014).

Phase 1: History taking. Client's major complaints, symptoms, and history of present illness were obtained. The client and the therapist jointly formed a hypothesis for the occurrence of symptoms and decided therapy targets.

Phase 2: Preparation. The theory, procedure and effect of the EMDR were explained. The therapist also provided techniques to control unpleasant feelings that occurred during the procedure.

Phase 3: Assessment. Each traumatic memory components, i.e., image, positive and negative beliefs, affect, and body sensation, was evaluated. Negative beliefs included negative feelings of self (e.g., I am helpless). Positive beliefs included thoughts that the client wished to have (e.g., I am valuable). Body sensations during target elicitation and simultaneous negative belief (e.g., choke) were examined.

Phase 4: Desensitization. The client focused on a target and the negative belief while pursuing a therapist's finger going left and right. A set consists of 24 rounds of left and right. Set repetitions reduce the negative feelings associated with the target.

Phase 5: Installation. The client elicited target images and the positive belief during the eye movements. This enhanced the client's positive belief.

Phase 6: Body scan. The client and the therapist jointly examined whether the client held the positive belief with the target or uncomfortable feelings remained. If they found a residual disturbance, then they performed the eye movements to target that residual disturbance to resolve the tension of the physical sensations.

Phase 7: Closure. The therapist confirmed the stability of the client's emotional state before closing the session.

Phase 8: Reassessment. At the beginning of the next session, the therapist assessed whether the uncomfortable feeling diminished or remained. If they still found a disturbance regarding the target, they again conducted desensitization and installation. If no negative feelings remained for the particular target, then they proceeded to the next target.

Phases 3 to 8 were repeated to process all the targets defined at Phase 1 one after the other. The therapy ended when all targets had been completed.

Phases 3 and 4 involved assessing the subjective intensity of disturbance or distress on a scale of 0 to 10 (Subjective Units of Disturbance [SUD] scale). Phase 5 took place after the SUD level came down to 0 or 1 at Phase 4.

Region-of-interest (ROI) analyses: overview

After the conventional random-effects (RFX) analysis, we next aimed to identify the relationships between the psychological assessment scores and neural activities. To achieve this goal, we conducted a series of ROI analyses. We first defined the center coordinates of ROIs using SPM regression analysis. We employed a psychological assessment score as the regressor and an estimate of Tooth Nar, Tooth Rem, Trauma Nar, or Trauma Rem as the variable. The following seven kinds of scores/subscales were employed: intrusion, avoidance, and hyperarousal from the IES-R-J, the DES-II score, and re-experiencing, avoidance, and dissociation from the RSDI. CAPS subscales were not used for this purpose (i.e., defining ROIs) because of overlap with other assessments as well as the similarity of the maps with those based on IES-R-J subscales. We included patients both before and after the treatment (Pt = Pt1 and Pt2) into the analyses with subscales of the IES-R-J, RSDI, and DES-II. We also conducted analyses with RSDI subscales using all participants (All = Pt1, Pt2, and Ct). Participants' division was included as a covariate. These combinations (i.e., Pt and All) were derived from a presumption that the brain activity would be correlated or "scaled" with psychological assessment scores regardless of participants' conditions (i.e., patient or not) and that a greater sample size would be better for detecting the correlations.

We consequently selected a total of eight center coordinates among local maxima in regression analyses with a threshold of $p<0.01$ (uncorrected) at the voxel-level and $p\leq 0.10$ at the cluster-level. These lenient thresholds were applied to include all ROIs considered to be related to the task paradigm. They were located in the bilateral hippocampus (HP), medial prefrontal cortex (mPFC), bilateral primary auditory cortex (A1), primary visual cortex (V1), left inferior frontal gyrus (IFG), and left superior temporal gyrus (STG). These ROIs were particularly selected from various peaks displayed on the maps under a specific consideration as follows. The HP is a well-known center of memory, which is crucially affected in PTSD. The mPFC is an important hub of the default mode network (DMN), which is also related to memory and affected in PTSD. The A1 and the V1 were selected because the corresponding primary brain functions, auditory and visual, might be affected in the patients' imagery system. The IFG and STG, both of the left hemisphere, were selected because they are the main language areas and might be affected in the patients' language system. Other areas of general interest, including the amygdala, which has often been reported in the previous PTSD research, and the posterior cingulate cortex, which is another important hub of the DMN, were not selected, because we failed to observe corresponding activation peaks in the maps based on the regression analysis mentioned above.

ROI masks were spheres made using the WFU PickAtlas toolbox (Tzourio-Mazoyer et al. 2002; Maldjian et al. 2003) with a center at one of these coordinates and a diameter of 20 mm. Contrast estimates within each ROI were individually averaged for each contrast (average

contrast estimate; ACE). The center coordinates of the ROIs are summarized later in **Supplementary Table S8**.

The following were examined with regard to the ROIs:

(1) Pearson's product-moment correlation coefficients of the ACEs with psychological assessment scores (**Supplementary Figures S3 and S4**).

(2) Repeated-measures ANOVAs using ACEs as the dependent variable (**Supplementary Table S9**).

(3) Correlation matrices among ACEs (**Figure 5A to C**; main text).

(4) Further analysis of the correlation matrices (**Supplementary Figure S5** and **Supplementary Table S10**).

(5) Specific analyses between Tooth Nar/Rem and Trauma Rem (**Supplementary Table S11 and S12**, and **Supplementary Figures S6 and S7**).

Activation maps by standard SPM analysis

Supplementary Figure S2 shows the activation maps generated during the conventional group study using SPM random-effects statistics, whereas **Supplementary Table S7** summarizes the peak coordinates of the activation clusters.

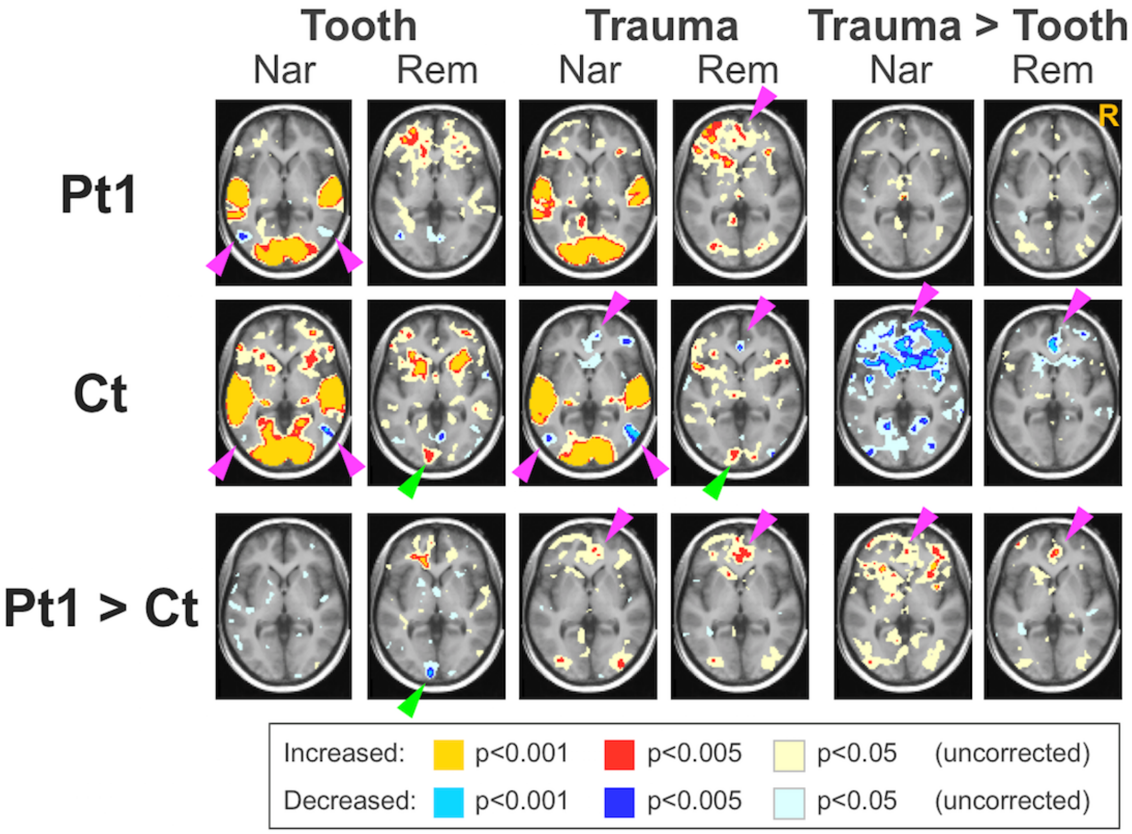

Supplementary Figure S2. Activation maps based on the random-effects group analysis. Activation maps at z=0 of the MNI space are superimposed on to the corresponding average T1-weighted images of participants (n=18). The green arrowheads indicate the primary visual cortex (V1). The magenta arrowheads indicate the DMN areas, including the medial prefrontal cortex (mPFC), as well as the extensions from the lateral parietal cortex. R: right.

Supplementary Table S7. Peak coordinates from standard group comparisons

Contrast	Direction	Coordinates			T	Anatomy
		x	y	z		
Tooth Rem	Pt1 > Ct	-16	36	-2	3.93	Lt Anterior Cingulate C
		-38	-16	28	3.87	Lt Precentral G
Tooth Rem	Ct > Pt1	40	-44	42	4.10	Rt Inferior Parietal Lobule
		-30	-2	54	4.03	Lt Medial Frontal G
		-54	-40	44	3.89	Lt Inferior Parietal Lobule
		-6	-96	-4	3.64	Lt V1
Trauma Nar	Pt1 > Ct	40	-64	-14	3.71	Rt Inferior Occipital G
		40	-82	-8	3.65	Rt Inferior Occipital G
		-30	-80	4	3.50	Lt Middle Occipital G
		-36	-64	-12	3.17	Lt Fusiform G
Trauma Rem	Pt1 > Ct	40	-78	38	4.10	Rt Middle Occipital G
		-2	38	2	3.91	Lt Anterior Cingulate C
Trauma Nar > Tooth Nar	Pt1 > Ct	34	34	-10	4.52	Rt IFG Orbital part
		-18	8	-8	4.34	Lt Putamen
		-16	-62	-16	4.29	Lt Culmen
		36	-42	-12	4.28	Rt Fusiform G
		-32	-52	-46	3.96	Lt Cerebellar Tonsil
		-22	42	-14	3.96	Lt Orbital G
		-54	-56	-8	3.52	Lt Inferior Temporal G
Trauma Rem > Tooth Rem	Pt1 > Ct	40	-78	38	3.84	Rt Middle Occipital G
		4	46	-2	3.25	Rt Anterior Cingulate C

Notes. Peak coordinates of clusters with $p < 0.005$ (uncorrected) for the voxel level and $p < 0.1$ (uncorrected) for the cluster level. Lt, left. Rt, right. C, cortex. G, gyrus.

Details of ROIs

The coordinates shown in **Supplementary Table S8** correspond to the local maxima obtained from SPM regression analysis with psychological assessment scores.

Supplementary Table S8. Summary of ROIs

ROI	Coordinates			Regression analysis				
	x	y	z	T	Group	Contrast	Assessment	Direction
Left HP	–22	–30	–14	4.07	Pt	Trauma Rem	IES-R-J hyperarousal	Positive
Right HP	34	–18	–16	3.6	All	Trauma Rem	RSDI dissociation	Positive
mPFC	0	46	10	4.05	All	Trauma Rem	RSDI dissociation	Positive
Left A1	–40	–26	4	3.75	Pt	Tooth Rem	DES-II	Positive
Right A1	42	–26	8	4.91	Pt	Tooth Rem	DES-II	Positive
V1	18	–72	0	4.69	Pt	Trauma Rem	IES-R-J avoidance	Positive
IFG	–42	12	12	5.23	Pt	Trauma Nar	RSDI avoidance	Negative
STG	–62	–40	6	3.55	Pt	Tooth Rem	IES-R-J intrusion	Positive

Notes. Coordinates from the Montreal Neurological Institute (MNI) template. T: t-value at the location. Group: participant groups included in regression analysis. Pt: Pt1 and Pt2. All: Pt1, Pt2, and Ct. Contrast: SPM contrast that the regression analysis used as data. Assessment: assessment subscale that the regression analysis included as a regressor. Direction: direction of the correlation. HP: hippocampus. mPFC: medial prefrontal cortex. A1: primary auditory cortex. V1: primary visual cortex. IFG: inferior frontal gyrus. STG: superior temporal gyrus.

Correlations between assessment scores and ROIs

We observed Pearson's product-moment correlation coefficients between assessment scores and contrast estimate values in ROIs. **Supplementary Figure S3** summarizes heatmap matrices of whole correlations, whereas **Supplementary Figure S4** shows the selected scatter diagrams with significant correlations. In the scatter diagrams, the statistical significance level is not always stringent, specifically in panels **A**, **H**, **I**, **K**, and **N**; this is because these diagrams (next to the section images), derived from the SPM regression analysis mentioned earlier and using the threshold used for the regression analysis ($p<0.01$, uncorrected, for the voxel-level), did not necessarily apply to all the voxels inside the ROIs, resulting in a weaker correlation between the ACE and the assessment score.

As described in the main text, we found that the bilateral HP presented both positive and negative correlations with the hyperarousal subscale of the IES-R-J in patients (**Fig. 4; Supplementary Fig. S4A to G**). We additionally found that the IFG had a negative correlation with the avoidance subscale of the RSDI during Trauma Nar (R=-0.681, $p<0.05$, n=9), but a positive correlation with the avoidance subscale of the IES-R-J during Tooth Nar (R=0.621, $p<0.10$, n=9) (**Supplementary Fig. S4N and O**).

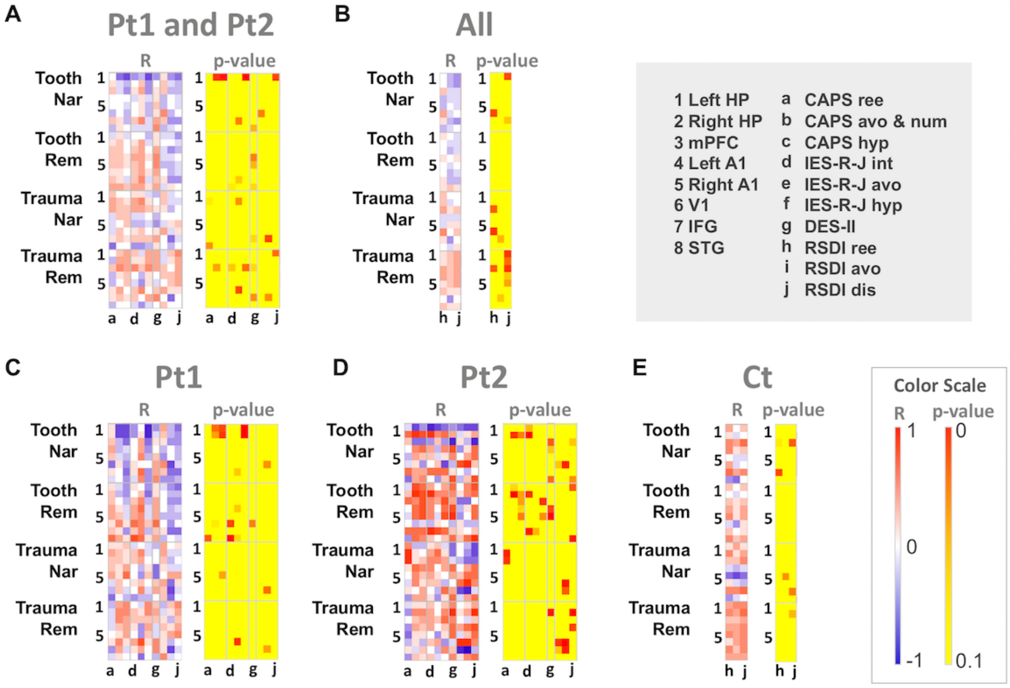

Supplementary Figure S3. Matrices of correlation coefficients (R) between average contrast estimates (ACEs) in ROIs and assessment scores (left side) and the p-values (right side). Matrices (**A**) for patients' first and second scans combined, (**B**) for all participants' scans combined, (**C**) for patients' first scans, (**D**) for patients' second scans, and (**E**) for controls' scans.

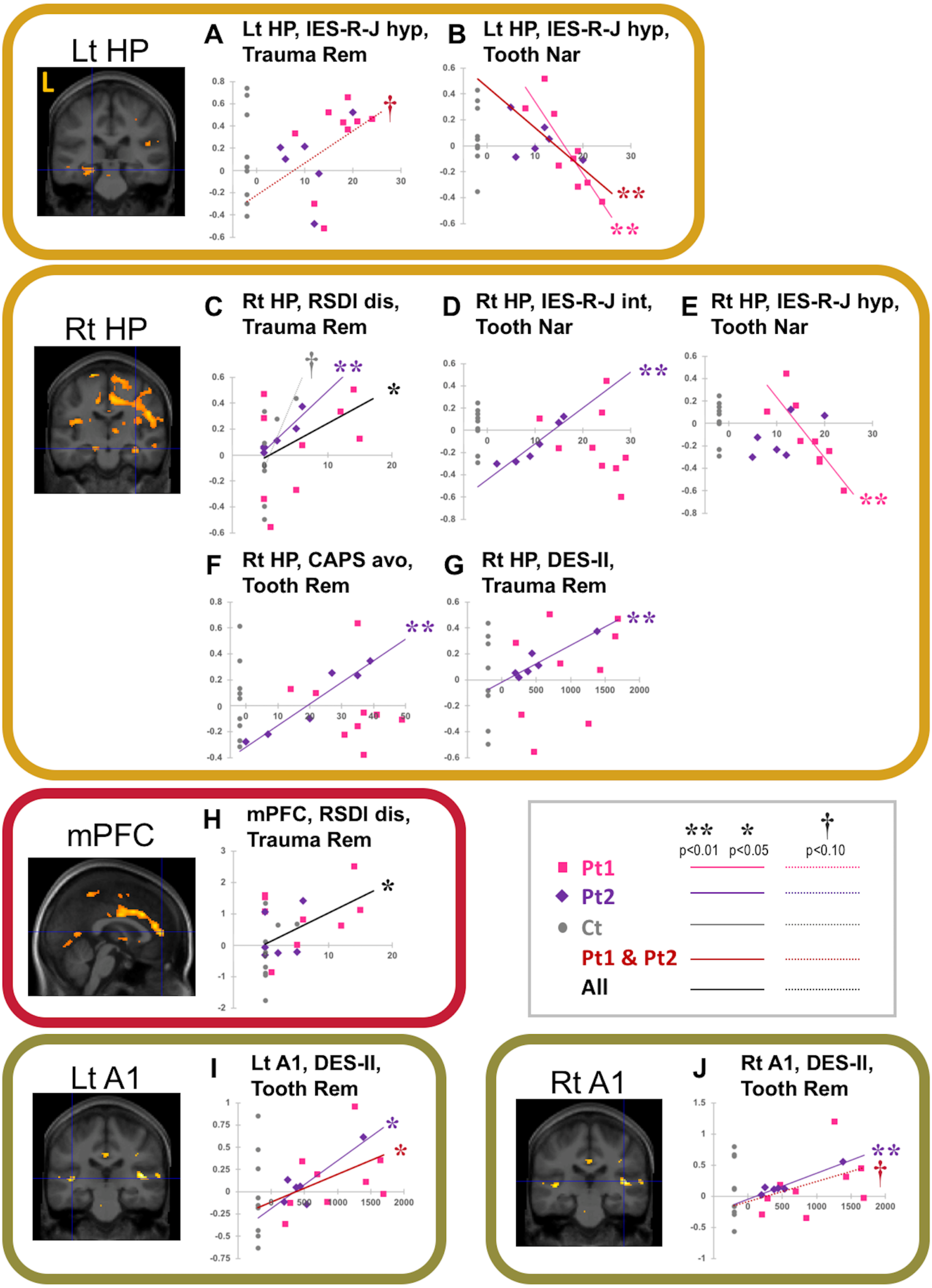
Lt HP
A Lt HP, IES-R-J hyp, Trauma Rem
B Lt HP, IES-R-J hyp, Tooth Nar
Rt HP
C Rt HP, RSDI dis, Trauma Rem
D Rt HP, IES-R-J int, Tooth Nar
E Rt HP, IES-R-J hyp, Tooth Nar
F Rt HP, CAPS avo, Tooth Rem
G Rt HP, DES-II, Trauma Rem
mPFC
H mPFC, RSDI dis, Trauma Rem
** p<0.01
* p<0.05
† p<0.10
Pt1
Pt2
Ct
Pt1 & Pt2
All
Lt A1
I Lt A1, DES-II, Tooth Rem
Rt A1
J Rt A1, DES-II, Tooth Rem

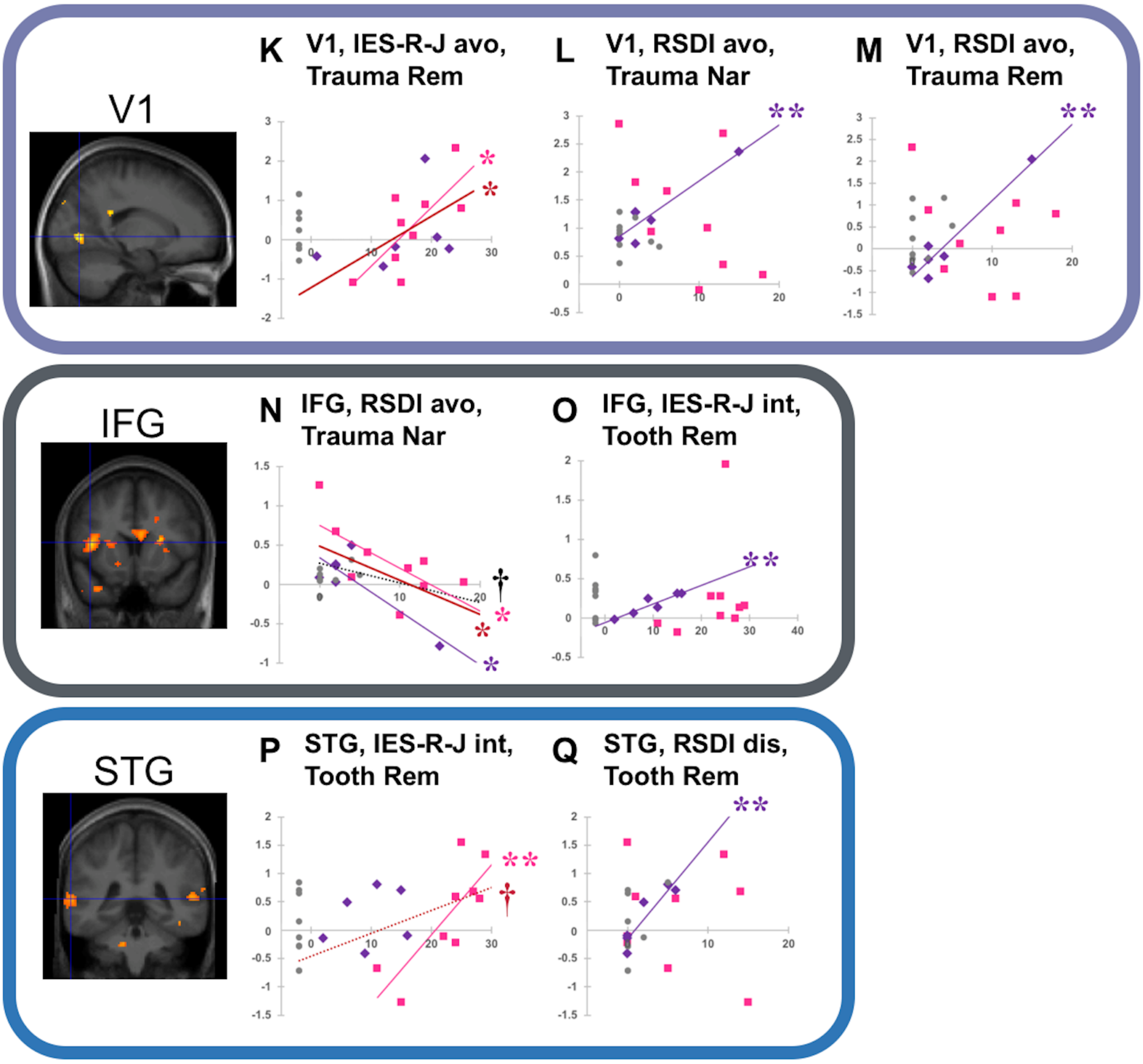

Supplementary Figure S4. Observation of remarked scatter diagrams for assessment scores at ROIs. The horizontal axis indicates an assessment subscale score, whereas the vertical axis indicates the average contrast estimate (ACE) value. Section images were generated by the respective SPM regression analyses in **Supplementary Table S8** ($p<0.01$, uncorrected), where hairline crosses indicate the local maxima. Scatter diagrams next to the section images (**A**, **C**, **H**, **I**, **J**, **K**, **N**, and **P**) are derived from the respective SPM regression analyses defining the ROIs (**Supplementary Table S8**). The other (**B**, **D**, **E**, **F**, **G**, **L**, **M**, **O**, and **Q**) are those selected based on the criterion of $p<0.01$ for at least one of the participant groups by a test of no correlation conducted for the ROI-subscale combination indicated in the header of the diagram; a linear prediction is plotted when the test of no correlation indicates a statistical significance. Lt: left. Rt: right. hyp: hyperarousal. dis: dissociation. int: intrusion. avo: avoidance. The gray circle indicates data by Ct; magenta square, Pt1; and purple diamond, Pt2. Linear prediction lines of the same color indicate the corresponding participant groups; additionally, the brown line indicates a prediction line for Pt1 and Pt2 combined; black, all Pt1, Pt2, and Ct combined.

A solid line indicates a linear prediction of $p<0.01$ (**) or $p<0.05$ (*) whereas a dashed line, of $p<0.10$ (†). For Ct with no assessment scores for a particular diagram, the ACE distribution is shown in the left side of the horizontal axis vertically.

ANOVA of activity estimates in ROIs

We aimed to capture features of patients (Pt1; 9 people), those recovered (Pt2; 6 people), and the matched controls (Ct; 9 people), and performed repeated-measures ANOVAs using the average contrast estimates (ACEs) within the ROIs as the dependent variable. As the sample size varied, we applied two ANOVA designs:

1. With three within-factors: group (Pt1, Pt2, and Ct; six people each), ROI (eight in total), and contrast conditions (Tooth Nar, Tooth Rem, Trauma Nar, and Trauma Rem; four in total).
2. With the same three within-factors except group (Pt1 and Ct; nine people each).

Both computed generalized omega squared (G.O.^2) to estimate the effect size, and used Holm's sequentially rejective Bonferroni procedure (alpha: 0.05) for the post hoc test (**Supplementary Table S9**).

We found significant main effects for ROI and contrast (ANOVA for Pt1, Pt2, and Ct: $F(7, 35)=14.500$, $p<0.000$ for ROI; $F(3, 15)=9.894$, $p<0.000$ for contrast; n=6 each. ANOVA for Pt1 and Ct: $F(7, 56)=11.316$, $p<0.000$ for ROI; $F(3, 24)=4.434$, $p<0.05$ for contrast; n=9 each) but not for group. Significant interaction effects were also found between ROI and contrast (ANOVA for Pt1, Pt2, and Ct: $F(21, 105)=18.375$, $p<0.000$, n=6 each. ANOVA for Pt1 and Ct: $F(21, 168)=11.641$, $p<0.000$, n=9 each); however, we failed to detect significant interaction effects with group.

Supplementary Table S9. ANOVA of activity estimates in ROIs

Supplementary Table S9-1. ANOVA for Pt1, Pt2 and Ct (6 people each)

Source	F	df	P	G.O.^2	Post hoc test
**Main effect**					
Group	1.803	2, 10	0.215	0.006	–
ROI	14.500	7, 35	0.000***	0.210	STG,LtA1>LtHP,mPFC,RtHP; RtA1>LtHP,RtHP
Contrast	9.894	3, 15	0.001***	0.119	TrNar>ToRem,TrRem; ToNar>ToRem
**Interaction**					
Group*ROI	1.368	14, 70	0.193	0.011	–
Group*Contrast	0.784	6,30	0.589	-0.007	–
ROI*Contrast	18.375	21, 105	0.000***	0.201	ToNar: STG,LtA1>IFG,LtHP,RtHP,mPFC; V1>RtHP,mPFC; RtA1>LtHP,RtHP,mPFC TrNar: STG,LtA1>IFG,LtHP,RtHP,mPFC;

					V1>RtHP,mPFC;<br>RtA1>LtHP,RtHP,mPFC<br>LtA1: TrNar,ToNar>TrRem>ToRem<br>RtA1: TrNar>ToRem,TrRem;<br>ToNar>ToRem<br>V1: ToNar,TrNar>ToRem,TrRem<br>STG: ToNar,TrNar>ToRem,TrRem
Group*ROI*Contrast	0.959	42, 210	0.549	-0.001	–

Supplementary Table S9-2. ANOVA for Pt1 and Ct (9 people each).

Source	F	df	P	G.O.^2	Post hoc test
**Main effect**					
Group	1.435	1, 8	0.265	0.002	–
ROI	11.316	7, 56	0.000***	0.125	STG,V1,LtA1,RtA1>LtHP,RtHP;<br>STG>LtA1,IFG; IFG>RtHP
Contrast	4.434	3, 24	0.013*	0.058	ToNar>ToRem
**Interaction**					
Group*ROI	2.001	7, 56	0.071 †	0.011	Pt1: STG,LtA1,RtA1>RtHP;<br>STG,LtA1>LtHP<br>Ct:<br>STG,V1,LtA1,RtA1>LtHP,RtHP;<br>STG,V1>IFG
Group*Contrast	0.717	3, 24	0.552	-0.004	–
ROI*Contrast	11.641	21, 168	0.000***	0.142	ToNar:<br>STG>LtA1,RtA1,IFG,mPFC,LtHP,<br>RtHP; V1,LtA1,RtA1>LtHP,RtHP;<br>V1,LtA1,RtA1>IFG<br>TrNar:<br>STG,V1,LtA1>IFG,LtHP,RtHP,mPF<br>C; RtA1>RtHP<br>LtA1: ToNar,TrNar>ToRem,TrRem<br>RtA1: ToNar>ToRem<br>V1: ToNar,TrNar>ToRem;<br>TrNar>TrRem<br>STG: TrNar,ToNar>TrRem,ToRem
Group*ROI*Contrast	2.085	21, 168	0.006**	0.013	(omit)

Notes. G.O.^2: generalized omega squared. Post hoc test: Holm's sequentially rejective Bonferroni procedure

(alpha=0.05). Names of ROIs follow **Supplementary Table S8**. Lt: left. Rt: right. ToNar: Tooth Nar. ToRem: Tooth Rem. TrNar: Trauma Nar. TrRem: Trauma Rem. ***: $p<0.001$. **: $p<0.01$. *: $p<0.05$. †: $p<0.10$.

Analysis of correlation matrices

We performed Jennrich's test (Jennrich 1970) and a one-way ANOVA to analyze matrices of correlation coefficients of average contrast estimates (ACEs) in ROIs between contrast conditions (**Fig. 5A to C** in the main text). Jennrich's test was to estimate the equality of matrices (Jennrich, 1970). We made several segmentations in the matrix (**Supplementary Fig. S5A**) and compared between and within participant groups (**Supplementary Table S10**). All comparisons indicated a complete difference between matrices. Chi-squares reflected the magnitude of the difference. Specifically, the difference between Tooth and Trauma was the greatest in Pt1 among the three groups (**Supplementary Table S10-2**).

To further examine the matrices, we performed a one-way ANOVA for each participant group. We tested differences among cells 1 to 4 using the correlation coefficients after a Fisher transformation by integrating ROIs (**Supplementary Fig. S5B**). We found a significant difference in Pt1 (F(3,252): 21.560, p < 0.000, G.O.^2: 0.194). The post hoc test indicated that correlations between Trauma Rem and Tooth Nar (cell 3 in **Supplementary Fig. S5B**) were the smallest, followed by those between Trauma Rem and Tooth Rem (cell 4), while those between Trauma Nar and Tooth Nar (cell 1) and those between Trauma Nar and Tooth Rem (cell 2) were the largest. By contrast, Pt2 showed no significant differences. Ct indicated a significant difference (F(3,252): 32.862, p < 0.000, G.O.^2: 0.272), where correlations between Trauma Rem and Tooth Rem (cell 4) were higher than all others. These results supported the group characteristics implicated in **Figure 5A to C** in the main text.

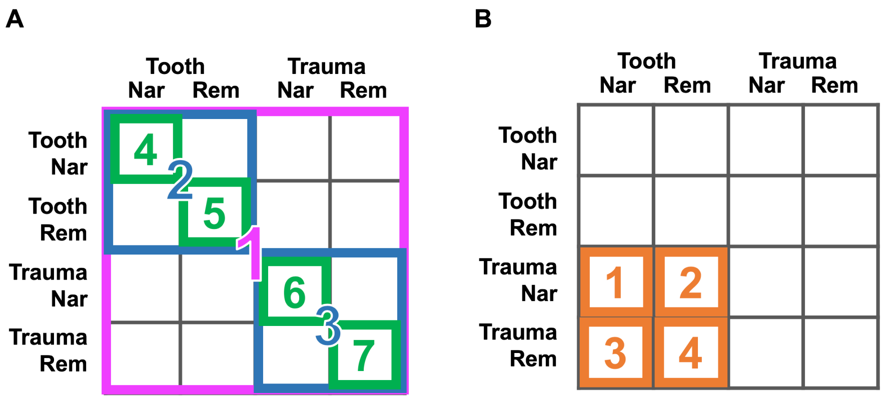

Supplementary Figure S5. Cells used in the matrix analyses. Each of the matrices (**A** and **B**) is a representation of a correlation matrix in **Figure 5 (A to C)**. (**A**) Cells used in Jennrich's test. (**B**) Cells used in ANOVA.

Supplementary Table S10. Jennrich's test for correlation matrices of average contrast estimates in ROIs

Supplementary Table S10-1. Between groups

	Matrix 1	Matrix 2	Chi-square	Matrix ID
Whole (matrix size 32 by 32, *df*=496)				
	Pt1	Ct1	21866.00	1
	Pt1	Pt2	4410.30	1
	Pt2	Ct1	103850.00	1
Task (matrix size 16 by 16, *df*=120)				
Tooth	Pt1	Ct1	174060.00	2
Tooth	Pt1	Pt2	11475.00	2
Tooth	Pt2	Ct1	1005.00	2
Trauma	Pt1	Ct1	204530.00	3
Trauma	Pt1	Pt2	34443.00	3
Trauma	Pt2	Ct1	33942.00	3
Block (matrix size 8 by 8, *df*=28)				
Tooth Nar	Pt1	Ct1	284.52	4
Tooth Nar	Pt1	Pt2	123.17	4
Tooth Nar	Pt2	Ct1	63.48	4
Tooth Rem	Pt1	Ct1	202.66	5
Tooth Rem	Pt1	Pt2	165.98	5
Tooth Rem	Pt2	Ct1	176.56	5
Trauma Nar	Pt1	Ct1	291.28	6
Trauma Nar	Pt1	Pt2	497.39	6
Trauma Nar	Pt2	Ct1	262.11	6
Trauma Rem	Pt1	Ct1	222.70	7
Trauma Rem	Pt1	Pt2	207.20	7
Trauma Rem	Pt2	Ct1	354.56	7

Supplementary Table S10-2. Within group

	Matrix 1	Matrix 2	Chi square	Matrix ID
Task (matrix size 16 by 16, *df*=120)				
Pt1	Tooth	Trauma	547680.00	2 vs. 3
Pt2	Tooth	Trauma	966.32	2 vs. 3
Ct	Tooth	Trauma	9087.60	2 vs. 3
Block (matrix size 8 by 8, *df*=28)				
Pt1	Tooth Nar	Tooth Rem	249.43	4 vs. 5
Pt1	Tooth Nar	Trauma Nar	158.59	4 vs. 6
Pt1	Tooth Nar	Trauma Rem	509.66	4 vs. 7
Pt1	Tooth Rem	Trauma Nar	250.63	5 vs. 6
Pt1	Tooth Rem	Trauma Rem	259.22	5 vs. 7
Pt1	Trauma Nar	Trauma Rem	280.50	6 vs. 7
Pt2	Tooth Nar	Tooth Rem	351.11	4 vs. 5
Pt2	Tooth Nar	Trauma Nar	506.02	4 vs. 6
Pt2	Tooth Nar	Trauma Rem	187.36	4 vs. 7
Pt2	Tooth Rem	Trauma Nar	554.62	5 vs. 6
Pt2	Tooth Rem	Trauma Rem	785.34	5 vs. 7
Pt2	Trauma Nar	Trauma Rem	559.21	6 vs. 7
Ct	Tooth Nar	Tooth Rem	143.87	4 vs. 5
Ct	Tooth Nar	Trauma Nar	65.57	4 vs. 6
Ct	Tooth Nar	Trauma Rem	120.97	4 vs. 7
Ct	Tooth Rem	Trauma Nar	70.55	5 vs. 6
Ct	Tooth Rem	Trauma Rem	177.41	5 vs. 7
Ct	Trauma Nar	Trauma Rem	80.83	6 vs. 7

Notes. Matrix ID: cell number indicated in **Supplementary Figure S5A**. All comparisons indicated $p<0.0001$.

Correlations in ROIs between Tooth Nar/Rem and Trauma Rem

We found intense negative correlations in the correlation maps of Pt1 between Tooth Nar and Trauma Rem as well as between Tooth Rem and Trauma Rem. To further characterize ROIs, we examined Spearman's rank correlation coefficient of each ROI between these contrasts of interest for Pt1. We detected intense negative correlations in the bilateral hippocampus (HP) as well as in the bilateral primary auditory cortex (A1) (**Supplementary Table S11**). Spearman's rank correlation coefficient was used because we were concerned about the effects of outliers.

Supplementary Table S11. Correlations in ROIs found in Pt1

	Tooth Nar and Trauma Rem			Tooth Rem and Trauma Rem		
	RS	P		RS	P	
Left HP	-0.867	0.005	**	-0.333	0.385	
Right HP	-0.617	0.086	†	-0.517	0.162	
mPFC	-0.033	0.948		-0.200	0.613	
Left A1	-0.983	0.000	***	-0.483	0.194	
Right A1	-0.933	0.001	***	-0.667	0.059	†
V1	0.500	0.178		0.683	0.050	†
IFG	0.083	0.843		0.167	0.678	
STG	-0.400	0.291		-0.133	0.744	

Notes. RS, Spearman's rank correlation coefficient. P, p-value. †, p<0.10. **, p<0.01. ***, p<0.001. Abbreviations for ROI names are the same as those in **Supplementary Table S8**.

Analysis of discrepancies between Tooth Nar/Rem and Trauma Rem

Discrepancies between contrast estimates of Tooth Nar/Rem and Trauma Rem were analyzed by focusing on the following three points. The first was to see whether remitted patients had a smaller discrepancy than other types. The second was to see whether patients (Pt1) had a greater discrepancy than others. The third was to see whether subjective disturbance (SUD) had a relationship with the discrepancy in patients.

Analysis methods

We examined not only bilateral HP and A1 ROIs, which showed negative correlations between Tooth Nar/Rem and Trauma Rem (**Supplementary Table S11**), but also peaks of negative correlations (**Fig. 6; Table 2**) in the same way; we extracted contrast estimates from spheres (diameter of 20 mm) with a center peak coordinate, computed the average contrast estimate, and analyzed the absolute values of the differences between conditions of interest. The main reason to conduct the latter examination for the negative peaks was that the center coordinates of the HP and A1 ROIs did not necessarily fit in the peaks of negative correlations (see coronal sections in **Fig. 7)**. The first and second points above were examined for both HP/A1 ROIs and negative peaks. The third was only examined for HP and A1 ROIs. We integrated data to conduct a one-way ANOVA to compare three levels of remitted, discontinued, and severe, or to compare the three levels of Pt1, Pt2, and Ct. We used t-test to compare between remitted and discontinued for Pt2.

Results and Discussion

For the first point above, the discrepancies in activity estimates of the HP and A1 **(Fig. 7A** to **D**) as well as those in peaks of negative correlations (**Supplementary Fig. S6**) apparently indicated that the remitted patients had a smaller discrepancy in general (**Supplementary Table S12**). In fact, the discrepancy seemed to increase with severity, i.e., from remitted, discontinued, to severe. For the second point, Pt1 indeed appeared to show a greater discrepancy between activity estimates than did Pt2 and Ct in general **(Fig. 7A** to **D; Supplementary Fig. S6; Supplementary Table S12**). As discussed in the main text, these results suggested that the discrepancy between activity estimates of the negative correlations reflected PTSD severity; the greater the discrepancy, the worse the symptoms.

By contrast, for the third point, the relationship between SUD and the activity discrepancy was not necessarily clear, except for the left HP (**Supplementary Fig. S7**). A possible explanation for the results might be that the patients' self-report of subjective disturbance could not objectively reflect the real severity of the symptoms.

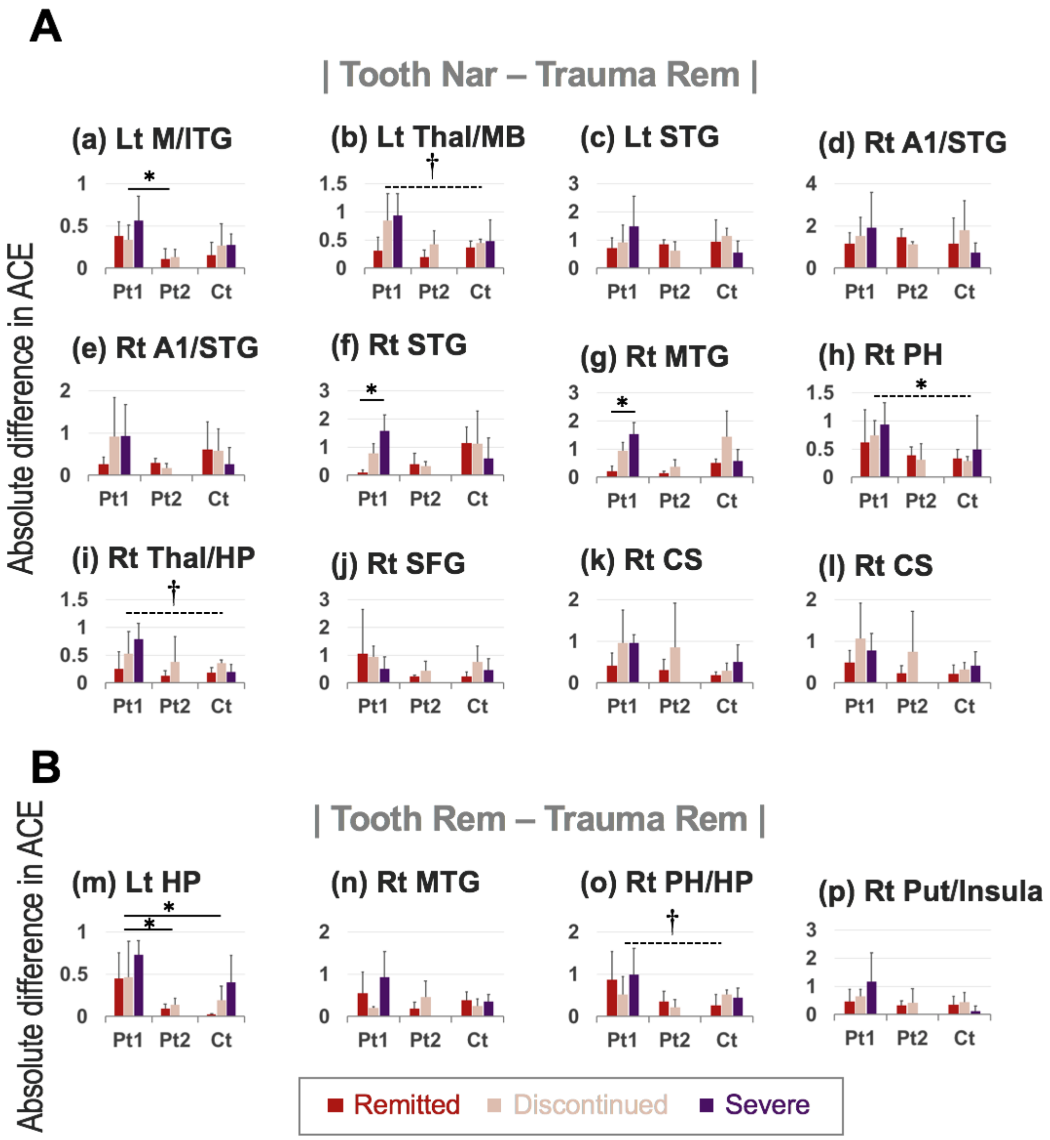

Supplementary Figure S6. Summary of discrepancies in activity estimates at the peaks of negative correlations labeled in **Table 2** in the main text. *, $p<0.05$. †, $p<0.10$. Statistical details are provided in **Supplementary Table S12**.

Supplementary Table S12. Summary of statistics for absolute difference between conditions

Supplementary Table S12-1. ROIs of left and right HP and A1

ROI	Contrast pair	Group	Comp.	Type	Statistics	Post hoc
Lt HP	Tooth Nar & Trauma Rem	All (N=24)	Pt1, Pt2, Ct	ANOVA	F=3.586, p=0.046*, G.O.^2=0.177	None
Lt HP	Tooth Nar & Trauma Rem	Pt1 (N=9)	Rem, Dis, Sev	ANOVA	F=9.082, p=0.015*, G.O.^2=0.642	Dis>Rem, Sev>Rem
Lt HP	Tooth Rem & Trauma Rem	All (N=24)	Pt1, Pt2, Ct	ANOVA	F=2.922, p=0.076 † , G.O.^2=0.138	None
Rt HP	Tooth Nar & Trauma Rem	All (N=24)	Pt1, Pt2, Ct	ANOVA	F=12.470, p=0.000***, G.O.^2=0.489	Pt1>Ct, Pt1>Pt2
Rt HP	Tooth Nar & Trauma Rem	Pt1 (N=9)	Rem, Dis, Sev	ANOVA	F=5.470, p=0.044*, G.O.^2=0.498	None
Rt HP	Tooth Rem & Trauma Rem	All (N=24)	Pt1, Pt2, Ct	ANOVA	F=9.269, p=0.001**, G.O.^2=0.408	Pt1>Pt2, Pt1>Ct
Lt A1	Tooth Nar & Trauma Rem	Pt2 (N=6)	Rem, Dis	t-test	t=3.112, df=3.833, p=0.038*	Rem>Dis
Rt A1	Tooth Rem & Trauma Rem	All (N=24)	Pt1, Pt2, Ct	ANOVA	F=2.694, p=0.091 † , G.O.^2=0.124	None
Four[*1] (4)	Both[*2] (2)	Pt1 (N=9*4*2)	Rem, Dis, Sev	ANOVA	F=6.868, p=0.002**, G.O.^2=0.140	Dis>Rem, Sev>Rem
Four[*1] (4)	Both[*2] (2)	All (N=24*4*2)	Pt1, Pt2, Ct	ANOVA	F=14.042, P=0.000***, G.O.^2=0.120	Pt1>Pt2, Pt1>Ct

Supplementary Table S12-2. Negative peaks labeled in **Table 2**

Peaks	Contrast pair	Group	Comp.	Type	Statistics	Post hoc
(a) Lt MTG/ITG	Tooth Nar & Trauma Rem	All (N=24)	Pt1, Pt2, Ct	ANOVA	F=5.890, p=0.009**, G.O.^2=0.290	Pt1>Pt2
(b) Lt MTG/ITG	Tooth Nar & Trauma Rem	All (N=24)	Pt1, Pt2, Ct	ANOVA	F=3.079, p=0.067 †, G.O.^2=0.148	None
(f) Rt STG/Pole	Tooth Nar & Trauma Rem	Pt1 (N=9)	Rem, Dis, Sev	ANOVA	F=10.714, p=0.011*, G.O.^2=0.683	Sev>Rem
(g) Rt MTG	Tooth Nar & Trauma Rem	Pt1 (N=9)	Rem, Dis, Sev	ANOVA	F=14.080, p=0.005**, G.O.^2=0.744	Sev>Rem
(h) Rt PH	Tooth Nar & Trauma Rem	All (N=24)	Pt1, Pt2, Ct	ANOVA	F=4.120, P=0.031*, G.O.^2=0.206	None
(i) Rt Thalamus/HP	Tooth Nar & Trauma Rem	All (N=24)	Pt1, Pt2, Ct	ANOVA	F=2.608, p=0.097 †, G.O.^2=0.118	None
(m) Lt HP	Tooth Rem & Trauma Rem	All (N=24)	Pt1, Pt2, Ct	ANOVA	F=6.949, p=0.005**, G.O.^2=0.332	Pt1>Pt2, Pt1>Ct
(o) Rt PH/HP	Tooth Rem & Trauma Rem	All (N=24)	Pt1, Pt2, Ct	ANOVA	F=3.860, p=0.037*, G.O.^2=0.193	None
All peaks*3 (16)	Depends on peak	Pt1 (N=9*16)	Rem, Dis, Sev	ANOVA	F=9.528, p=0.000***, G.O.^2=0.106	Sev>Dis>Rem
All peaks*3 (16)	Depends on peak	All (N=24*16)	Pt1, Pt2, Ct	ANOVA	F=16.189, p=0.000***, G.O.^2=0.073	Pt1>Pt2, Pt1>Ct

Notes. Only statistics with significant differences are reported. Comp., targets of comparison made; Rem, remitted; Dis, discontinued; Sev, severe. *1, integration of the examined four examined ROIs, i.e., the left and right HP and the left and right A1. *2, both Tooth Nar & Trauma Rem and Tooth Rem & Trauma Rem. *3, all 16 peaks labeled in **Table 2** (a to p). ***, p<0.001. **, p<0.01. *, p<0.05. †, p<0.10.

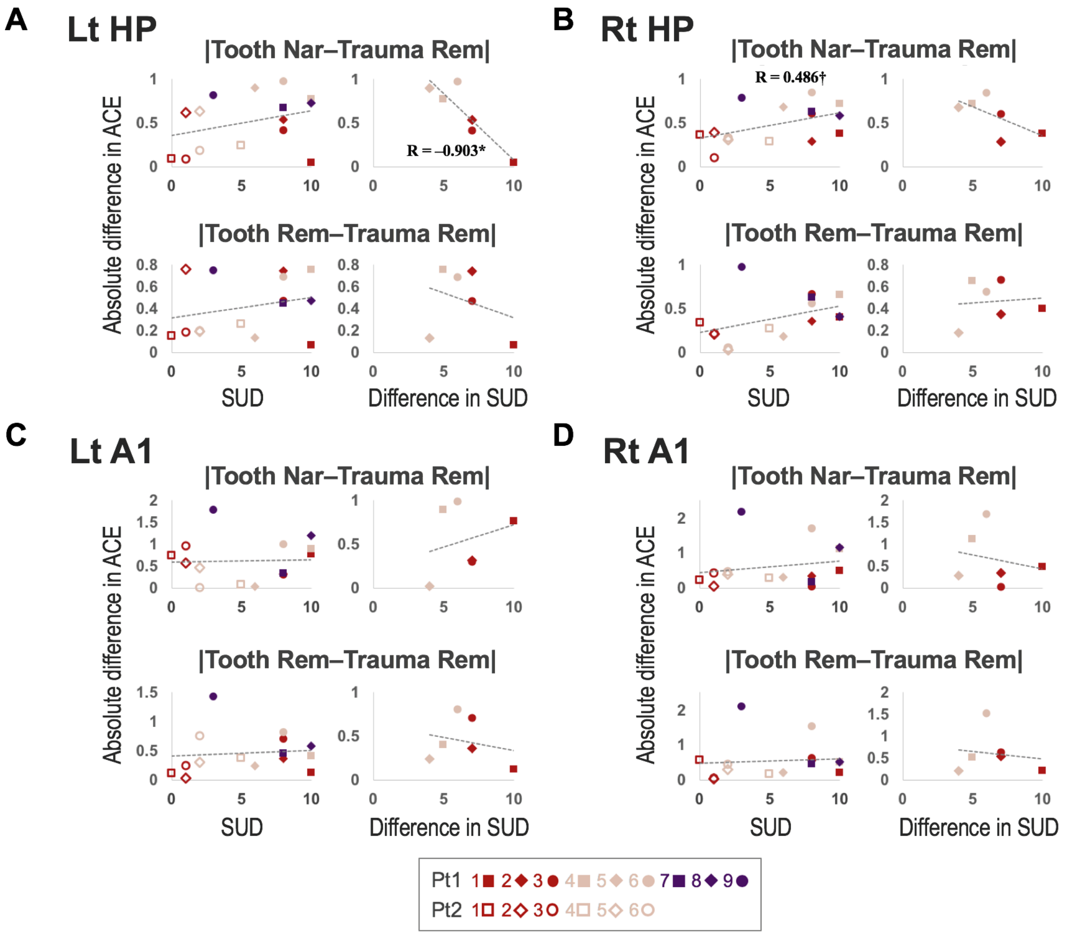

Supplementary Figure S7. Scatter graphs showing relationships between subjective unit of disturbance (SUD) and discrepancy of activity estimates in ROIs of the bilateral HP and A1. “Difference in SUD” indicates the changes between values before and after EMDR therapy. The upper two panels for the left hippocampus (A) are redundantly displayed for readers’ convenience although already shown in **Figure 7E**. The equations indicate Pearson’s product-moment correlation coefficient. *, $p<0.05$. †, $p<0.10$.

SPM comparison between Trauma Rem and Tooth Nar

We conducted an SPM computation to compare the magnitude of activity estimates between Trauma Rem and Tooth Nar. **Supplementary Figure S8** displays coronal sections showing the differential activities in Pt1 near the left and right hippocampi.

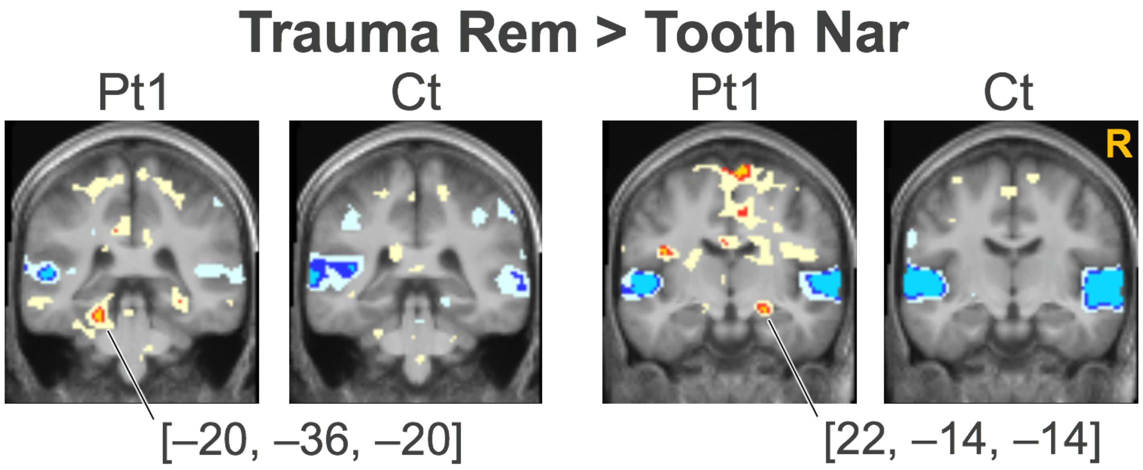

Supplementary Figure S8. Activation maps of Trauma Rem versus Tooth Nar. N=18. Coordinates of activation peaks near the hippocampus and/or the parahippocampal gyrus. The left two panels show the coronal section of y=–36, whereas the right two show y=–14. R: right.

References

Bremner JD, Narayan M, Staib LH, Southwick SM, McGlashan T, Charney DS. 1999. Neural correlates of memories of childhood sexual abuse in women with and without posttraumatic stress disorder. The American Journal of Psychiatry. 156:1787-1795.

Britton JC, Phan KL, Taylor SF, Fig LM, Liberzon I. 2005. Corticolimbic blood flow in posttraumatic stress disorder during script-driven imagery. Biological Psychiatry. 57:832-840.

Dahlgren MK, Laifer LM, VanElzakker MB, Offringa R, Hughes KC, Staples-Bradley LK, Dubois SJ, Lasko NB, Hinojosa CA, Orr SP, Pitman RK, Shin LM. 2018. Diminished medial prefrontal cortex activation during the recollection of stressful events is an acquired characteristic of PTSD. Psychological Medicine. 48:1128-1138.

Driessen M, Beblo T, Mertens M, Piefke M, Rullkoetter N, Silva-Saavedra A, Reddemann L, Rau H, Markowitsch HJ, Wulff H, Lange W, Woermann FG. 2004. Posttraumatic stress disorder and fMRI activation patterns of traumatic memory in patients with borderline personality disorder. Biological Psychiatry. 55:603-611.

Fani N, Ashraf A, Afzal N, Jawed F, Kitayama N, Reed L, Bremner JD. 2011. Increased neural response to trauma scripts in posttraumatic stress disorder following paroxetine treatment: A pilot study. Neuroscience Letters. 491:196-201.

Frewen PA, Lanius RA, Dozois DJ, Neufeld RW, Pain C, Hopper JW, Densmore M, Stevens TK. 2008. Clinical and neural correlates of alexithymia in posttraumatic stress disorder. Journal of Abnormal Psychology. 117:171-181.

Hopper JW, Frewen PA, Sack M, Lanius RA, van der Kolk BA. 2007a. The responses to Script-Driven Imagery Scale (RSDI): Assessment of state posttraumatic symptoms for psychobiological and treatment research. Journal of Psychopathology and Behavioral Assessment. 29:249-268.

Hopper JW, Frewen PA, van der Kolk BA, Lanius RA. 2007b. Neural correlates of reexperiencing, avoidance, and dissociation in PTSD: symptom dimensions and emotion dysregulation in responses to script-driven trauma imagery. Journal of Traumatic Stress. 20:713-725.

International Society for the Study of Trauma and Dissociation. 2011. Guidelines for treating dissociative identity disorder in adults, third revision. Journal of Trauma & Dissociation. 12:115-187.

Jennrich RI. 1970. An asymptotic $\chi 2$ test for the equality of two correlation matrices. Journal of the American Statistical Association. 65:904-912.

Lanius RA, Bluhm R, Lanius U, Pain C. 2006. A review of neuroimaging studies in PTSD: heterogeneity of response to symptom provocation. Journal of Psychiatric Research. 40:709-729.

Lanius RA, Frewen PA, Girotti M, Neufeld RW, Stevens TK, Densmore M. 2007. Neural correlates of trauma script-imagery in posttraumatic stress disorder with and without comorbid major depression: a functional MRI investigation. Psychiatry Research. 155:45-56.

Lanius RA, Williamson PC, Bluhm RL, Densmore M, Boksman K, Neufeld RW, Gati JS, Menon RS. 2005. Functional connectivity of dissociative responses in posttraumatic stress disorder: a functional magnetic resonance imaging investigation. Biological Psychiatry. 57:873-884.

Lanius RA, Williamson PC, Boksman K, Densmore M, Gupta M, Neufeld RW, Gati JS, Menon RS. 2002. Brain activation during script-driven imagery induced dissociative responses in PTSD: a functional magnetic resonance imaging investigation. Biological Psychiatry. 52:305-311.

Lanius RA, Williamson PC, Densmore M, Boksman K, Gupta MA, Neufeld RW, Gati JS, Menon RS. 2001. Neural correlates of traumatic memories in posttraumatic stress disorder: a functional MRI investigation. The American Journal of Psychiatry. 158:1920-1922.

Lanius RA, Williamson PC, Hopper J, Densmore M, Boksman K, Gupta MA, Neufeld RW, Gati JS, Menon RS. 2003. Recall of emotional states in posttraumatic stress disorder: an fMRI investigation. Biological Psychiatry. 53:204-210.

Levin P, Lazrove S, van der Kolk B. 1999. What psychological testing and neuroimaging tell us about the treatment of Posttraumatic Stress Disorder by Eye Movement Desensitization and Reprocessing. Journal of Anxiety Disorders. 13:159-172.

Lindauer RJ, Booij J, Habraken JB, van Meijel EP, Uylings HB, Olff M, Carlier IV, den Heeten GJ, van Eck-Smit BL, Gersons BP. 2008. Effects of psychotherapy on regional cerebral blood flow during trauma imagery in patients with post-traumatic stress disorder: a randomized clinical trial. Psychological Medicine. 38:543-554.

Ludäscher P, Valerius G, Stiglmayr C, Mauchnik J, Lanius RA, Bohus M, Schmahl C. 2010. Pain sensitivity and neural processing during dissociative states in patients with borderline personality disorder with and without comorbid posttraumatic stress disorder: a pilot study. Journal of Psychiatry & Neuroscience. 35:177-184.

Maldjian JA, Laurienti PJ, Kraft RA, Burdette JH. 2003. An automated method for neuroanatomic and cytoarchitectonic atlas-based interrogation of fMRI data sets. NeuroImage. 19:1233-1239.

Malejko K, Abler B, Plener PL, Straub J. 2017. Neural correlates of psychotherapeutic treatment of post-traumatic stress disorder: a systematic literature review. Frontiers in Psychiatry. 8:85.

Mickleborough MJ, Daniels JK, Coupland NJ, Kao R, Williamson PC, Lanius UF, Hegadoren K, Schore A, Densmore M, Stevens T, Lanius RA. 2011. Effects of trauma-related cues on pain processing in posttraumatic stress disorder: an fMRI investigation. Journal of

Psychiatry & Neuroscience. 36:6-14.

Moher D, Hopewell S, Schulz KF, Montori V, Gotzsche PC, Devereaux PJ, Elbourne D, Egger M, Altman DG. 2010. CONSORT 2010 explanation and elaboration: updated guidelines for reporting parallel group randomised trials. BMJ. 340:c869.

Nakagawa S, Cuthill IC. 2007. Effect size, confidence interval and statistical significance: a practical guide for biologists. Biological Reviews of the Cambridge Philosophical Society. 82:591-605.

Osuch EA, Benson B, Geraci M, Podell D, Herscovitch P, McCann UD, Post RM. 2001. Regional cerebral blood flow correlated with flashback intensity in patients with posttraumatic stress disorder. Biological Psychiatry. 50:246-253.

Pagani M, Högberg G, Salmaso D, Nardo D, Sundin O, Jonsson C, Soares J, Aberg-Wistedt A, Jacobsson H, Larsson SA, Hällström T. 2007. Effects of EMDR psychotherapy on 99mTc-HMPAO distribution in occupation-related post-traumatic stress disorder. Nuclear Medicine Communications. 28:757-765.

Peres JF, Foerster B, Santana LG, Fereira MD, Nasello AG, Savoia M, Moreira-Almeida A, Lederman H. 2011. Police officers under attack: resilience implications of an fMRI study. Journal of Psychiatric Research. 45:727-734.

Peres JF, Newberg AB, Mercante JP, Simão M, Albuquerque VE, Peres MJ, Nasello AG. 2007. Cerebral blood flow changes during retrieval of traumatic memories before and after psychotherapy: a SPECT study. Psychological Medicine. 37:1481-1491.

Piefke M, Pestinger M, Arin T, Kohl B, Kastrau F, Schnitker R, Vohn R, Weber J, Ohnhaus M, Erli HJ, Perlitz V, Paar O, Petzold ER, Flatten G. 2007. The neurofunctional mechanisms of traumatic and non-traumatic memory in patients with acute PTSD following accident trauma. Neurocase. 13:342-357.

Rauch SL, van der Kolk BA, Fisler RE, Alpert NM, Orr SP, Savage CR, Fischman AJ, Jenike MA, Pitman RK. 1996. A symptom provocation study of posttraumatic stress disorder using positron emission tomography and script-driven imagery. Archives of General Psychiatry. 53:380-387.

Sack M, Cillien M, Hopper JW. 2012. Acute dissociation and cardiac reactivity to script-driven imagery in trauma-related disorders. European Journal of Psychotraumatology. 3.

Shapiro F. 1995. Eye movement desensitization and reprocessing, basic principles, protocols and procedures. New York: Guilford Press.

Shapiro F. 2001. Eye movement desensitization and reprocessing, basic principles, protocols and procedures (2nd ed.). New York: Guilford Press.

Shapiro F. 2014. The role of eye movement desensitization and reprocessing (EMDR) therapy in medicine: addressing the psychological and physical symptoms stemming from adverse life experiences. The Permanente Journal. 18:71-77.

Shin LM, McNally RJ, Kosslyn SM, Thompson WL, Rauch SL, Alpert NM, Metzger LJ, Lasko

NB, Orr SP, Pitman RK. 1999. Regional cerebral blood flow during script-driven imagery in childhood sexual abuse-related PTSD: A PET investigation. The American Journal of Psychiatry. 156:575-584.

Shin LM, Orr SP, Carson MA, Rauch SL, Macklin ML, Lasko NB, Peters PM, Metzger LJ, Dougherty DD, Cannistraro PA, Alpert NM, Fischman AJ, Pitman RK. 2004. Regional cerebral blood flow in the amygdala and medial prefrontal cortex during traumatic imagery in male and female Vietnam veterans with PTSD. Archives of General Psychiatry. 61:168-176.

Thome J, Terpou BA, McKinnon MC, Lanius RA. 2020. The neural correlates of trauma-related autobiographical memory in posttraumatic stress disorder: A meta-analysis. Depression and Anxiety. 37:321-345.

Thomaes K, Dorrepaal E, Draijer N, Jansma EP, Veltman DJ, van Balkom AJ. 2014. Can pharmacological and psychological treatment change brain structure and function in PTSD? A systematic review. Journal of Psychiatric Research. 50:1-15.

Tzourio-Mazoyer N, Landeau B, Papathanassiou D, Crivello F, Etard O, Delcroix N, Mazoyer B, Joliot M. 2002. Automated anatomical labeling of activations in SPM using a macroscopic anatomical parcellation of the MNI MRI single-subject brain. NeuroImage. 15:273-289.

Whalley MG, Kroes MC, Huntley Z, Rugg MD, Davis SW, Brewin CR. 2013. An fMRI investigation of posttraumatic flashbacks. Brain and Cognition. 81:151-159.
